# Reconstruction of Large Femoral Segmental Defects in Conflict-Related Trauma Using Non-vascularized Fibular and Iliac Crest Grafts: A Case Series

**DOI:** 10.7759/cureus.117328

**Published:** 2026-10-01

**Authors:** Laith Hseinat, Ahmad Almigdad, Majdala Al-Bataineh, Ghayth Al Adaileh, Osama Abu Zaid, Ashraf Albadaineh, Moayad Telfah, Faris Alzuheir, Hidar Al Soudi

**Affiliations:** 1 Department of Orthopaedics, Jordanian Royal Medical Services, Amman, JOR; 2 Faculty of Medicine, Yarmouk University, Irbid, JOR; 3 Orthopaedics and Traumatology, Jordanian Ministry of Health, Amman, JOR; 4 Department of Orthopaedics, Jordanian Royal Medical services, Amman, JOR

**Keywords:** bone reconstruction, conflict-related trauma, femoral nonunion, iliac crest bone grafts, limb-salvage, non-vascularized fibular graft, segmental bone defect

## Abstract

Introduction/objectives

Large post-traumatic femoral segmental defects with established nonunion represent a challenging reconstructive problem, particularly in conflict-affected and resource-limited environments where access to microsurgical reconstruction and prolonged staged procedures may be limited. Non-vascularized fibular grafting (NVFG) combined with autologous iliac crest bone grafting (ICBG) may provide both structural and biological support for reconstruction. This case series describes the clinical experience with this combined approach in patients with large femoral segmental defects following conflict-related trauma.

Methods

A case series of eight patients with femoral nonunion and segmental bone loss was conducted at the Jordanian Field Hospital in southern Gaza (Khan Younis) between June and September 2024. Patients were identified at presentation with established nonunion and segmental bone defects and underwent reconstruction using combined NVFG and autologous ICBG. Definitive reconstruction was performed as a single procedure in most patients, whereas staged management was undertaken when required for infection control. Clinical and radiographic follow-up were retrospectively reviewed, with outcomes assessed from routine postoperative clinical and radiographic follow-up for 13-18 months. The primary clinical outcome was radiographic progression to union, with additional assessment of complications, limb function, and need for revision surgery.

Results

The eight patients included six males and two females, with a mean age of 27.9 years (range, 12-40 years). Seven injuries resulted from blast trauma and one from a gunshot injury. Four patients had Gustilo type IIIC injuries, three had type IIIA injuries, and one had a closed injury. Segmental bone defects ranged from 6 to 24 cm, with five patients having defects exceeding 10 cm. Six patients achieved primary union, one achieved functional union with a residual cortical gap, and one achieved union after revision fixation following implant failure associated with premature weight bearing. Mean time to radiographic union was 7.3 months (range, 6-9 months). Four patients (50%) experienced complications, including implant failure requiring revision, residual limb-length discrepancy, and residual cortical deficiency.

Conclusion

Reconstruction using NVFG combined with autologous ICBG may represent a feasible option for large post-traumatic femoral segmental defects in conflict-affected and resource-limited settings. The technique provides structural support and biological augmentation without the microsurgical complexity of vascularized bone grafting. In this small case series, it offered a practical limb-salvage strategy in a high-demand environment, with staged infection management used when clinically required.

## Introduction

Nonunion is generally defined as failure of a fracture to progress toward healing over an expected period, with assessment based on clinical and radiographic findings. Union is generally defined clinically by painless, full- or partial-weight bearing and radiographically by the presence of bridging callus across at least three cortices on orthogonal radiographs [1]. The risk of nonunion varies depending on fracture characteristics, injury severity, fixation method, and patient-related factors. Factors such as open fractures, infection, smoking, obesity, and increasing injury severity have been associated with an increased risk of nonunion [2]. Recent data from conflict-related trauma care in Gaza further highlight this burden, where femur fractures constituted the most common injury pattern (36.5%), frequently associated with blast and gunshot mechanisms, extensive soft-tissue damage, and complex open injuries. Despite relatively low early complication rates, functional recovery remained limited, with only 30.1% of patients regaining independent ambulation at three months, underscoring the substantial disability associated with these injuries and the need for effective reconstructive strategies in resource-limited and conflict settings [3]. In the setting of war-related injuries and complex open femoral fractures, the risk of nonunion is further increased due to extensive contamination, impaired vascular supply, and disruption of the biological environment essential for bone healing [4].

War-related trauma in Gaza has resulted in a substantial burden of complex musculoskeletal injuries, with open femoral fractures associated with extensive soft tissue damage and neurovascular compromise representing a particularly challenging subset. This situation places considerable pressure on an already constrained healthcare system. Management is frequently limited to damage-control interventions due to delayed presentation and restricted availability of definitive surgical care. As a consequence, there is a high incidence of complications, including infection, joint stiffness, and nonunion, often converting potentially reconstructable injuries into complex limb salvage problems [5,6].

Management of femoral nonunion associated with segmental bone loss requires a broad spectrum of reconstructive modalities, including autologous bone grafting, vascularized and non-vascularized fibular grafts (NVFGs), distraction osteogenesis, allograft reconstruction, and staged approaches such as the Masquelet technique, which enhances graft incorporation through the development of an induced vascularized membrane [7,8]. Although multiple fixation strategies are available, biological enhancement remains fundamental, particularly in the context of high-energy trauma, infection, and open fractures, where mechanical stability alone is insufficient to achieve osseous union. Available fixation strategies include locking plates, intramedullary fixation, external fixation, and circular fixation, with the choice depending on defect location, bone quality, soft-tissue condition, and the reconstructive strategy [9]. Iliac crest bone graft (ICBG) remains a well-established source of autologous bone graft but is limited by donor-site morbidity and graft availability in large defects [10]. Fibular grafts are an established reconstructive option for segmental bone defects. Vascularized fibular grafts offer biological advantages but require microsurgical expertise, whereas NVFGs provide a simpler reconstructive alternative when combined with cancellous autograft [11].

In resource-constrained conflict environments such as Gaza, a considerable proportion of surgical care is provided by short-term visiting surgeons, which frequently limits access to staged reconstruction protocols and microsurgical techniques [5]. Consequently, there is a need for simpler, reliable, and reproducible single-stage reconstructive strategies.

This case series includes eight patients with femoral nonunion and segmental bone loss treated using non-vascularized fibular strut grafts combined with autologous ICBG. The series describes the application of this combined reconstructive approach to large femoral segmental defects, including defects exceeding 10 cm, in a conflict-affected setting characterized by delayed presentation and limited access to advanced staged and microsurgical reconstruction. Definitive reconstruction was performed as a single procedure in most patients, whereas staged management was required in the setting of active infection. Clinical and radiographic outcomes were subsequently assessed through routine postoperative follow-up.

## Materials and methods

Study design and patient selection

We conducted a retrospective case series of eight patients with established femoral nonunion and segmental bone defects who presented to the Jordanian Field Hospital in southern Gaza (Khan Younis) and underwent reconstructive treatment by the same operating surgeon between June and September 2024. This period represents the period of presentation and treatment at our field hospital rather than the dates of the original injuries. The original injuries occurred at different times before presentation to our team, and most patients had received initial fracture stabilization and/or other surgical treatment at healthcare facilities elsewhere. The interval from injury to presentation and definitive reconstruction therefore varied among patients and is detailed in the individual case presentations and Table 1. Information regarding the initial injury and previous treatment was obtained from available referral documentation, medical records, and patient or family history; where complete records were unavailable, only reliably documented information was reported.

**Table 1:** Patient demographics

Case	Age	Sex	Mechanism	Fracture Type	Defect (cm)	Fibular graft length (cm)
1	19	Male	Blast injury	Gustilo IIIA	21	26
2	27	Male	Blast injury	Gustilo IIIC	19	27
3	39	Male	Blast injury	Gustilo IIIC	11	17
4	12	Male	Blast injury	Gustilo IIIC	7	12
5	40	Female	Blast injury	Closed fracture	24	27
6	39	Male	Blast injury	Gustilo IIIC	15	21
7	33	Male	Gunshot	Gustilo IIIA	6	11
8	14	Female	Blast injury	Gustilo IIIA	6	13

Patients were included when they presented with an established femoral nonunion associated with a segmental bone defect and were considered suitable for reconstruction using combined NVFG and autologous ICBG. Suitability for this reconstructive strategy was determined according to the clinical presentation, characteristics and extent of the bone defect, soft-tissue condition, infection status, and available resources. At presentation, the available clinical records and previous treatment were reviewed, and patients underwent clinical, radiographic, and laboratory assessment before definitive reconstruction. The pre-referral condition and relevant previous procedures are described in the individual case presentations to the extent that reliable information was available. Definitive reconstruction was performed as a single procedure in seven patients, whereas one patient with an infected nonunion required staged management for infection control before definitive reconstruction.

This retrospective case series was based on clinical and radiographic outcomes documented during routine postoperative follow-up, which ranged from 13 to 18 months. The study was descriptive in nature, and no comparative group was included.

Preoperative assessment and infection evaluation

Patients underwent clinical and radiographic assessment before definitive reconstruction, including evaluation of the nonunion, segmental bone loss, limb alignment and length, soft-tissue condition, and evidence of infection. Laboratory evaluation, when available, included complete blood count, erythrocyte sedimentation rate (ESR), and C-reactive protein (CRP). In patients with suspected infection, intraoperative deep tissue specimens were obtained for microbiological culture.

In cases initially managed with external fixation, the fixator was removed before definitive reconstruction, and reconstruction was deferred until the pin sites had adequately healed and closed, typically over approximately 2-4 weeks. This interval was related to pin-site and soft-tissue healing and did not represent treatment of pin-site infection. Infection status was assessed separately based on clinical findings and, when indicated, inflammatory markers and intraoperative deep tissue cultures.

Calcium and vitamin D supplementation was provided routinely as part of perioperative supportive management; however, serum calcium and 25-hydroxyvitamin D levels could not be measured because the required laboratory facilities were not available in the field-hospital setting.

In patients with previous femoral arterial injury or vascular reconstruction, preoperative vascular assessment included Doppler ultrasonography and consultation with a vascular surgeon before ipsilateral fibular harvest. The feasibility and potential risks of fibular harvest were assessed on an individual basis.

Surgical technique

A common reconstructive technique was used, with case-specific variations according to the clinical presentation, defect characteristics, fixation requirements, vascular status, and implant availability. Due to limited access to hygiene resources in the field-hospital setting, patients were admitted one day before surgery and underwent full-body antiseptic bathing with chlorhexidine and soap on the evening before and morning of surgery. Intraoperative skin preparation included shaving and antisepsis of the recipient site, fibular donor site, and iliac crest using Sterillium or 70% isopropyl alcohol. Standard sterile techniques were maintained throughout. Intravenous tranexamic acid was administered perioperatively as a blood-conservation measure because of limited availability of blood products in the conflict-affected setting.

A lateral approach to the femur was used. The nonunion site was exposed, and fibrotic and nonviable tissue was excised until viable bleeding bone was encountered. The segmental defect was measured after completion of debridement, when the final extent of bone loss had been established. Limb length and alignment were assessed by comparison with the contralateral limb and intraoperative fluoroscopy.

The medullary canal was initially opened using a drill bit and subsequently enlarged gradually using a rigid DHS reamer until sufficient space was available to accommodate the selected fibular graft. No fixed reamer diameter was used because the canal dimensions varied according to the individual femoral anatomy and the selected graft. Reaming was performed slowly with saline irrigation to limit heat generation. Where necessary, the fibular graft was trimmed to obtain a close fit within the prepared canal.

The NVFG was inserted into the host medullary canal at both ends of the defect, with approximately 2-3 cm of intramedullary engagement where anatomically feasible, with the objective of obtaining stable intramedullary purchase and maximizing host-graft contact. In cases with exceptionally large defects, limited limb shortening was accepted when necessary to facilitate graft seating and improve mechanical stability. Graft position, alignment, and host-graft engagement were assessed by direct visualization and fluoroscopy before definitive fixation.

The fixation construct was selected according to the location and extent of the defect, local anatomy, mechanical requirements, and available implants. Fixation included long distal or proximal femoral locking compression plates (LCP), straight LCP, or dynamic condylar screw (DCS) constructs as described for the individual cases.

Autologous ICBG was harvested and used for biological augmentation. Where required, structural iliac crest bone blocks were placed around and at the graft-host interface, particularly in wider metaphyseal regions, to improve graft seating and mechanical support. Additional cancellous ICBG was packed around the graft-host junctions to enhance biological incorporation. The wound was closed in layers over a suction drain, which was generally maintained for 48-72 hours.

Fibular graft harvesting

Fibular grafts were harvested using either an open or percutaneous technique according to the clinical circumstances and surgeon preference. Care was taken to preserve adequate proximal and distal fibular segments and to protect the surrounding soft tissues and neurovascular structures.

For open harvesting, a lateral approach was used with careful dissection to the fibula. The osteotomy sites were identified under fluoroscopic guidance, and subperiosteal dissection was performed using a periosteal elevator. A malleable retractor was used to protect the surrounding soft tissues during drilling, and the osteotomies were completed with an osteotome. The graft was subsequently mobilized and removed using a Kocher clamp.

For percutaneous harvesting, an approximately 3-cm incision was used to expose the fibular shaft. Subperiosteal dissection was performed using a periosteal elevator, with fluoroscopic localization of the osteotomy sites. A malleable retractor was used to protect the surrounding soft tissues during drilling, and the graft was mobilized after release of the relevant soft-tissue attachments.

In patients requiring long fibular grafts, approximately 5-6 cm of fibula was preserved proximally and distally where anatomically feasible. The exact preoperative total fibular length was not routinely measured or documented; graft lengths reported in Table 1 represent the actual harvested graft lengths.

Assessment of union and clinical outcomes

Patients were assessed clinically and radiographically during follow-up. Clinical assessment included pain at the reconstruction site, progression of weight bearing, limb function, range of motion of adjacent joints, and complications. Radiographic assessment was performed using serial plain radiographs. Radiographic union was considered when progressive bridging and consolidation were present across at least three of the four cortices on orthogonal radiographs, together with compatible clinical evidence of healing. CT was obtained selectively when plain radiographs were inconclusive or when there was persistent clinical concern regarding incomplete union; it was not routinely performed in all patients.

Clinical and radiographic assessments were performed by the treating orthopedic team during follow-up. Independent blinded radiographic assessment and formal interobserver reliability analysis were not performed because of the field-hospital setting and the descriptive nature and small size of the series.

Functional outcomes were assessed clinically, including weight-bearing ability, ambulation, and range of motion of adjacent joints. Standardized patient-reported outcome measures such as AOFAS, FAOS, or equivalent validated scores were not systematically collected because of the logistical constraints of the conflict-affected, high-volume field-hospital environment. This represents a limitation of the present series.

Postoperative complications were defined as adverse events occurring following the reconstructive procedure that required additional treatment or represented a clinically relevant adverse outcome, including mechanical failure, residual limb-length discrepancy, or residual structural deficiency. Pre-existing stiffness that was treated during the index reconstruction was not considered a postoperative complication.

Postoperative rehabilitation

Postoperatively, patients began range-of-motion and bed-based exercises, followed by bed-to-chair mobilization. Toe-touch weight bearing was maintained for approximately 6-12 weeks, depending on the stability of the reconstruction and early radiographic evidence of healing. Once early radiographic signs of healing were observed, patients were progressed to partial weight bearing, generally until approximately 20 weeks. Thereafter, weight bearing was advanced as tolerated, with progression to unrestricted activity individualized according to radiographic consolidation, clinical symptoms, limb stability, and functional recovery.

In Case 8, premature progression to unrestricted weight bearing before adequate radiographic consolidation was followed by implant bending and subsequent revision fixation at another hospital after completion of the field-hospital mission.

Follow-up and reporting

Follow-up was performed by the treating surgical teams during subsequent missions and included clinical and radiographic evaluation. The individual follow-up duration ranged from 13 to 18 months, with longer follow-up available for selected patients as specifically described in the individual case presentations.

The manuscript was reviewed with reference to established reporting recommendations for case series and individual case descriptions, including the PROCESS and CARE frameworks, to improve the completeness and transparency of reporting.

Ethical considerations

The study was reviewed and approved by the institutional ethics committee. Written consent was obtained for publication of clinical details and images. For patients younger than 18 years, written consent was obtained from a parent or legal guardian for publication of their clinical details and images. For the pediatric patients, consent was obtained from the parent or legal guardian.

## Results

Clinical and functional outcomes

Union was achieved in six patients following the index reconstruction, with radiographic union occurring at a mean of 7.3 months (range, 6-9 months). Case 5 demonstrated progressive healing and functional union at 12 months, although a residual medial cortical gap measuring approximately 2-4 cm remained; therefore, complete radiological union was not considered to have been achieved. Case 8 subsequently achieved union 10 months after second-stage definitive reconstruction following implant failure after premature unrestricted weight bearing. Overall, all eight patients ultimately achieved either radiographic union or functional healing. At the latest follow-up, all patients were able to bear full weight without assistive devices, except that Case 8 had initially progressed to unrestricted weight bearing before implant failure and revision fixation. Knee range of motion ranged from 0-90° to 0-130° among patients, with Case 5 demonstrating a range of -10° to 100°. Documented postoperative complications occurred in four patients (50%), consisting of limb shortening of approximately 3 cm in Case 1 and 4 cm in Case 4, a residual medial cortical gap in Case 5, and implant failure requiring revision fixation in Case 8. No postoperative complications were documented in Cases 2, 3, 6, or 7 (Table 2).

**Table 2:** Clinical Outcomes *Case 5 demonstrated functional healing at 12 months, with bridging/consolidation across three of four cortices and a persistent medial cortical gap of approximately 2-4 cm. Complete radiographic union was not achieved; therefore, 12 months represents the time to documented functional healing rather than complete radiographic union. **Final radiographic consolidation occurred approximately 10 months after the initial definitive reconstruction, following revision fixation for implant failure. ROM, range of motion

Case	Union Status	Time to Union (months)	Weight Bearing	Knee ROM (Normal 0-140 degrees)	Complications
1	Achieved	8	Full weight bearing without assistive devices	0-130	Shortening (3 cm)
2	Achieved	7	Full weight bearing without assistive devices	0-120	None
3	Achieved	6	Full weight bearing without assistive devices	0-90	None
4	Achieved	6	Full weight bearing without assistive devices	0-130	Shortening (4 cm)
5	Functional union	12*	Full weight bearing without assistive devices on last follow-up at 18 months	-10 to 100	Residual medial cortical gap (2-4 cm); functional healing despite incomplete radiographic union
6	Achieved	8	Full weight bearing without assistive devices	0-100	None
7	Achieved	9	Full weight bearing without assistive devices	0-130	None
8	Union after revision fixation	10	Early unrestricted weight bearing prior to failure	0-130	Implant failure requiring revision fixation**

Case presentation

Case 1

Case 1 describes a 19-year-old previously healthy male patient who sustained a blast injury in December 2023, resulting in an open mid-shaft fracture of the right femur with associated entry and exit wounds. Based on the available historical clinical documentation, referral records, and contemporaneous gross photographs reviewed by our team, the initial injury was classified retrospectively as Gustilo-Anderson type IIIA. There was no associated neurovascular injury. Initial management included wound debridement and stabilization using an external fixator. Seven months later, he presented to the Jordanian Field Hospital with persistent pain, inability to bear weight on the affected limb, and a shortened right lower extremity. The wounds had healed without clinical evidence of infection. Clinical examination demonstrated full hip range of motion, while knee examination revealed a 30° loss of flexion with full extension. Plain radiographs demonstrated an established femoral shaft nonunion with a persistent segmental gap and no appreciable callus formation (Figure 1). Laboratory investigations showed a complete blood count within normal limits, an erythrocyte sedimentation rate of 30 mm/hour, and negative C-reactive protein, with no clinical or laboratory evidence of active infection.

**Figure 1:**
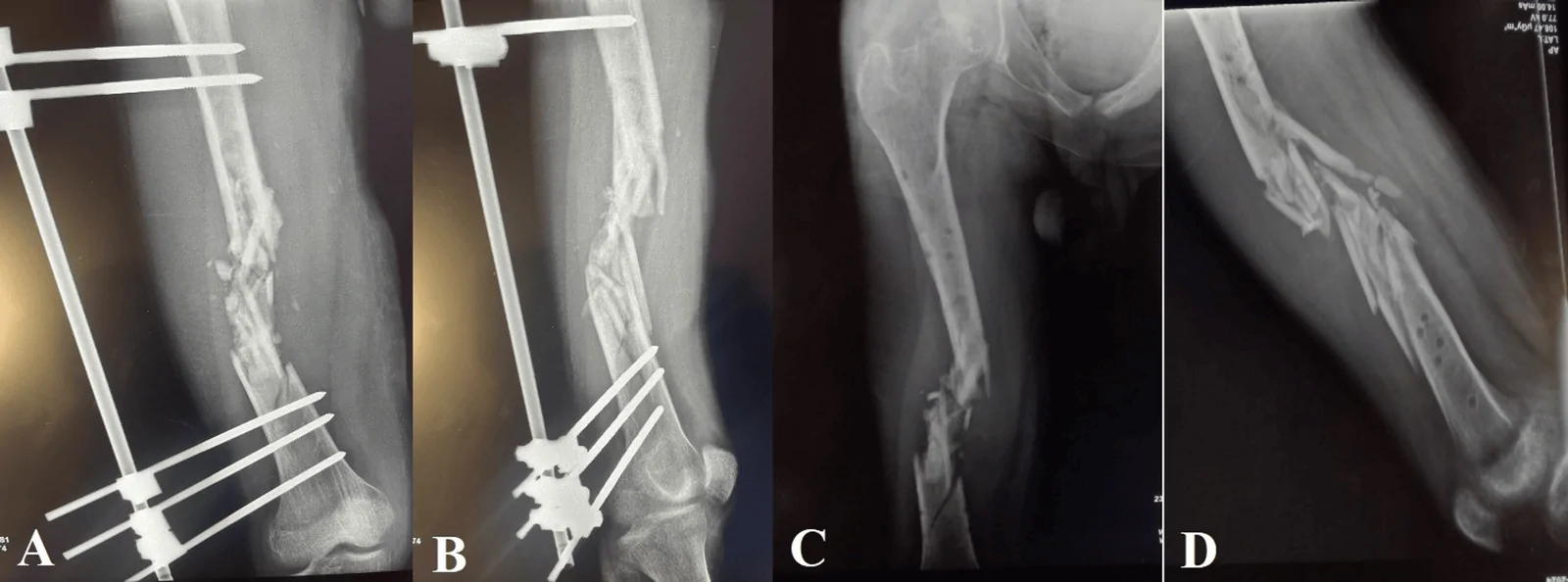
Presentation X-rays (A+B) Initial presentation AP and lateral view X-rays of the right femur showing established fracture nonunion with an obvious bone gap following external fixation. (C+D) Preoperative AP and lateral X-ray views after removal of the external fixator, demonstrating the persistent nonunion and bone defect.

Following exclusion of infection, definitive reconstruction was planned. Intraoperatively, all necrotic and nonviable bone was resected until viable bleeding bone was reached, resulting in a 21-cm segmental defect. Because of the exceptional size of the defect and the relatively long ipsilateral fibula, a 26-cm ipsilateral NVFG was harvested. The graft was inserted into the host medullary canals at both ends to obtain maximal feasible intramedullary purchase, with approximately 2-3 cm of graft seated within the host canal at each end where anatomically feasible. The proximal end was well docked within the medullary canal, while the wider distal metaphyseal canal was additionally supported with an iliac crest bone-graft block to improve graft seating and stability. A limited degree of limb shortening was accepted to facilitate secure graft engagement and maximize host-graft contact. Autologous ICBG was also placed at the graft-host junctions between the fixation plate and fibular graft. Graft position and alignment were assessed by direct examination and fluoroscopy before definitive stabilization using a long distal femoral LCP (Figure 2). No intraoperative complications occurred.

**Figure 2:**
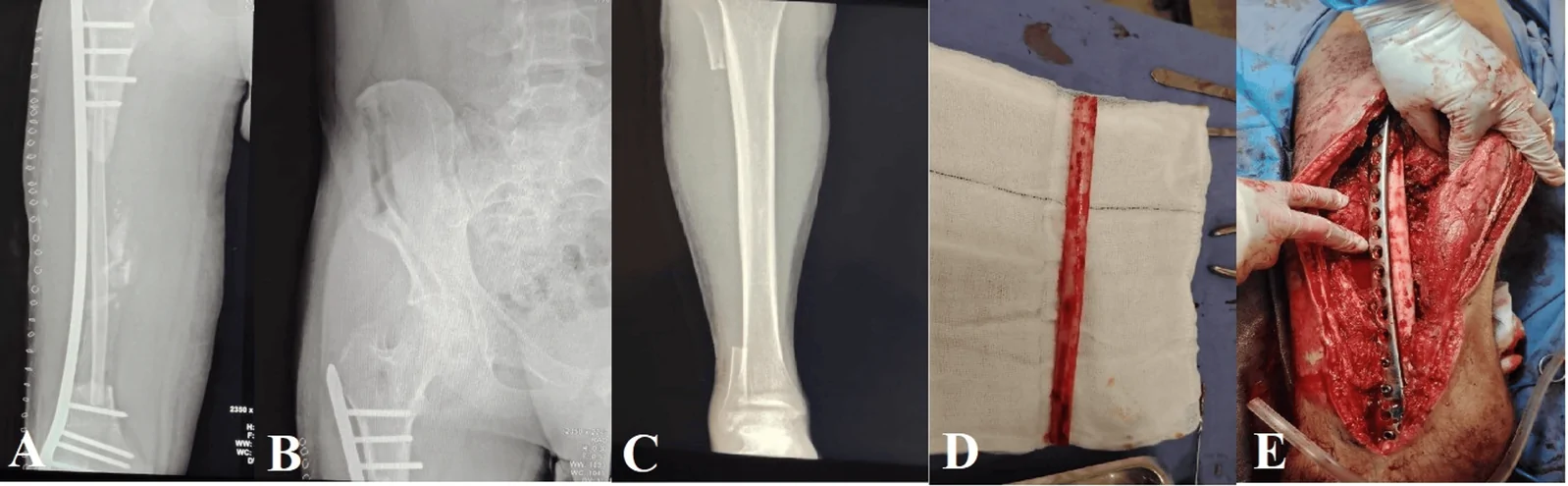
Gross intraoperative images and initial postoperative X-rays (A) Initial postoperative AP femur X-ray view showing the final NVFG with the application of iliac crest blocks in the final femur gap after debridement and excision of the dead bone, with fixation of the fracture with a distal femur LCP and restoration of the final length and alignment. (B) Postoperative AP iliac X-ray view showing the donor site of the right iliac crest. (C) Postoperative AP view of the right leg showing the donor site of the NVFG. (D) Intraoperative clinical gross photograph showing the final length of the NVFG. (E) Intraoperative clinical photograph showing the intramedullary placement of the NVFG with fixation with a distal femur LCP plate. AP: anteroposterior; NVFG: non-vascularized fibular graft; LCP: locking compression plate

Postoperatively, the patient was mobilized with protected weight bearing. Serial clinical and radiological follow-up demonstrated progressive healing, with bridging callus formation observed at three months and progressive osteointegration thereafter. At eight months postoperatively, sufficient radiological healing was achieved, allowing progression to full, unprotected weight bearing. Union was defined radiographically by bridging callus across at least three cortices. At the latest follow-up, 13 months after surgery, complete union was achieved with no evidence of infection, implant failure, or refracture (Figure 3). The patient was fully weight bearing and demonstrated good functional recovery, with knee range of motion from 0° to 130°. A residual limb-length discrepancy of approximately 3 cm was noted. No donor-site morbidity related to fibular graft harvesting was reported.

**Figure 3:**
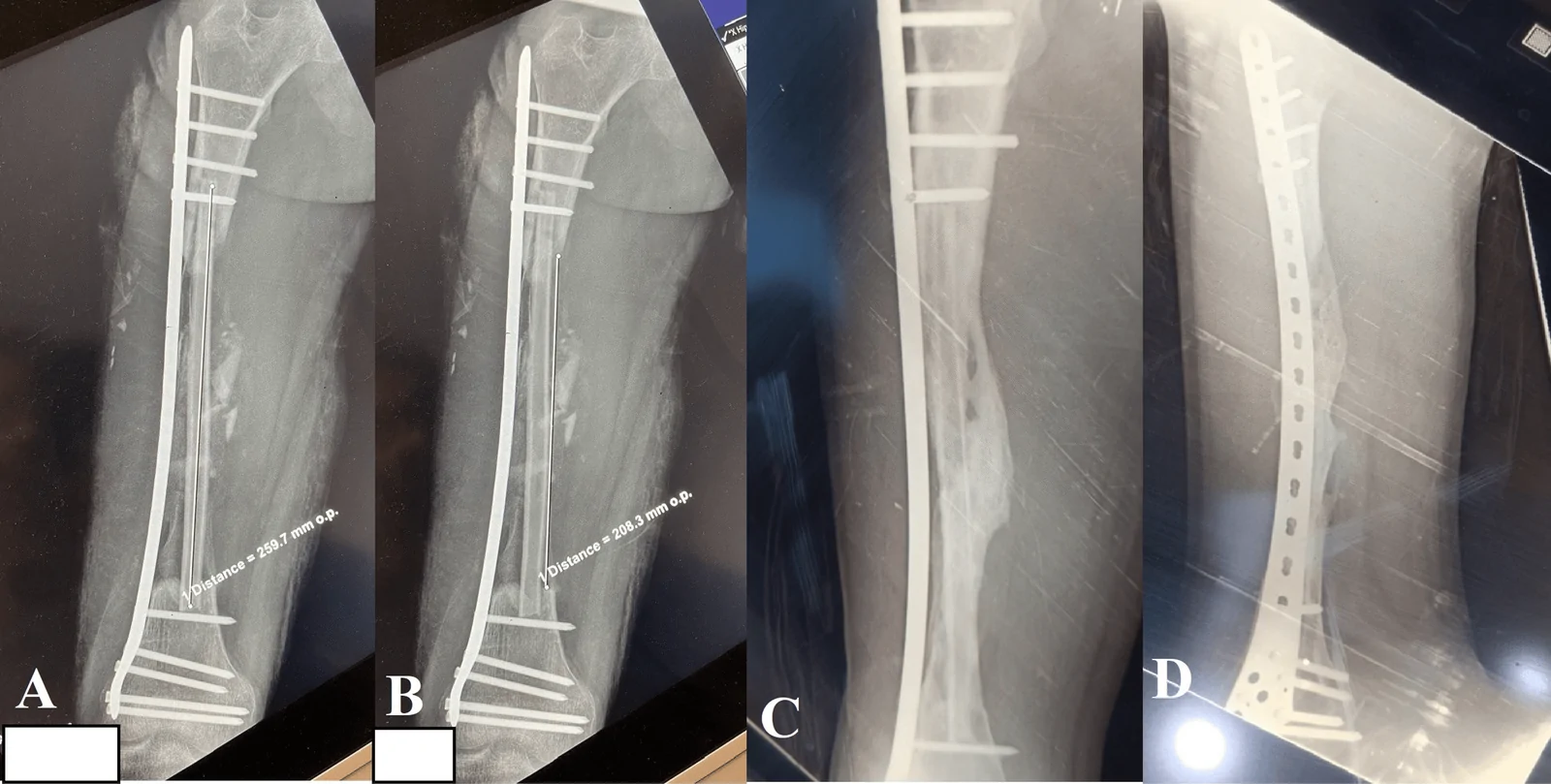
X-rays showing gap and fibular graft length with 4-week and 13-month follow-up Four-week follow-up X-rays in AP view showing the fibular graft length (A) and the gap length (B). Thirteen-month follow-up in AP view (C) and lateral (D) X-ray views showing complete healing of the fracture site and fibular osteointegration. AP: anteroposterior

Case 2

Case 2 involves a 27-year-old male patient who sustained a blast injury in November 2023, resulting in a Gustilo-Anderson type IIIC open fracture of the right femur associated with a right femoral artery injury. Initial management included surgical debridement, femoral artery bypass, and fracture stabilization using an external fixator. Ten months later, he presented to the Jordanian Field Hospital with persistent pain, inability to bear weight on the affected limb, and limb shortening. The wounds had healed without clinical evidence of infection. Hip range of motion was preserved, while knee examination demonstrated stiffness. Radiographs confirmed an established femoral nonunion with a persistent segmental gap and no significant callus formation (Figure 4). Laboratory investigations demonstrated a complete blood count within normal limits, an erythrocyte sedimentation rate of approximately 30 mm/hour, and negative C-reactive protein, with no clinical or laboratory evidence of active infection. Following exclusion of infection, single-stage reconstruction was performed. Necrotic and nonviable bone was resected until viable bleeding bone was reached, resulting in a 19-cm defect. Reconstruction was achieved using a 27-cm ipsilateral NVFG combined with autologous ICBG, with the graft positioned at the reconstruction site and supplemented with ICBG at the graft-host junctions. Stabilization was achieved using a distal femoral LCP. Manipulation under anesthesia was performed during the same procedure to address knee stiffness, achieving approximately 100° of knee flexion intraoperatively (Figure 5). No intraoperative complications occurred.

**Figure 4:**
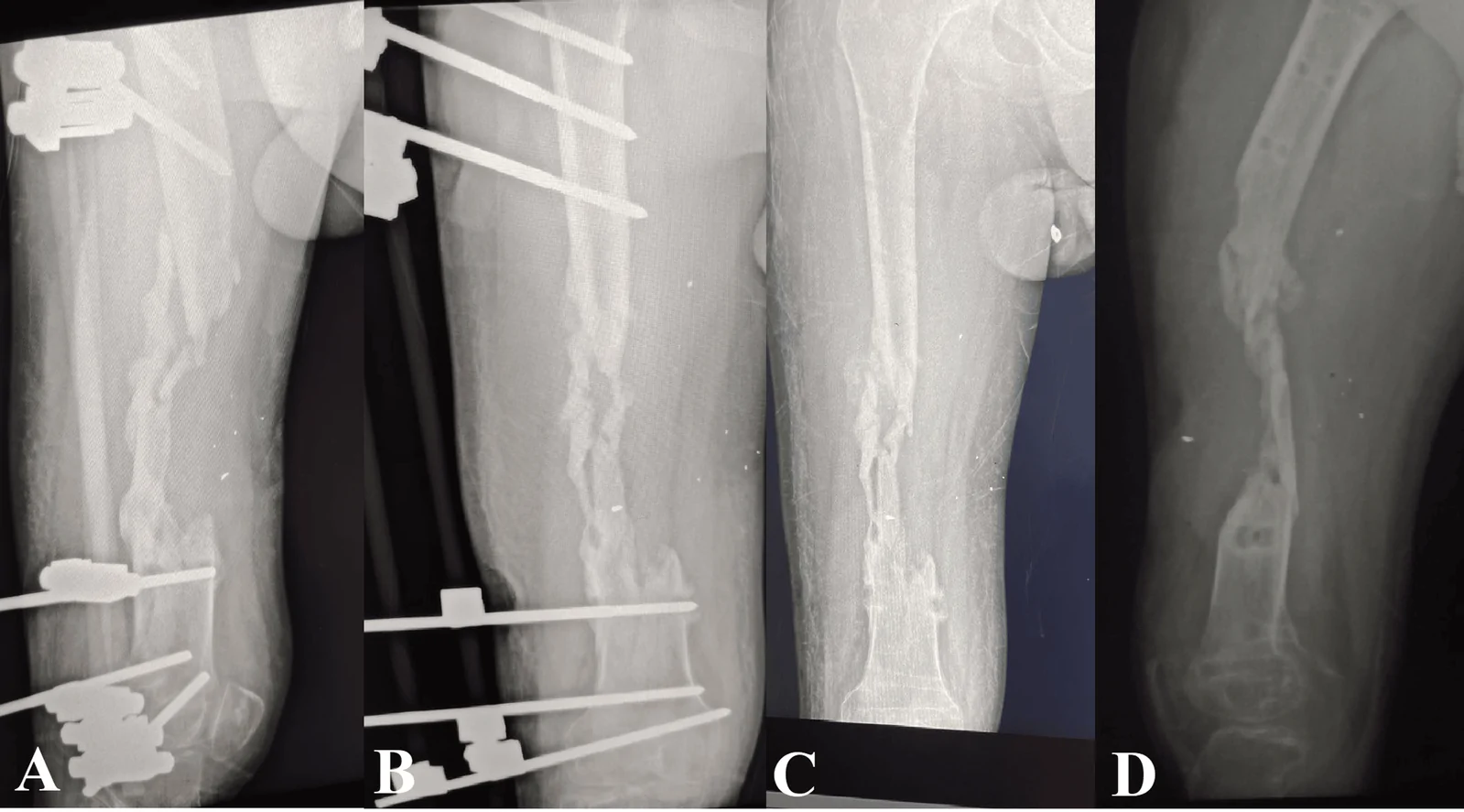
Initial presentation and preoperative X-rays (A+B) Initial AP and lateral X-ray views of the right femur demonstrating established Gustilo type IIIC fracture nonunion following external fixation, with associated bone loss. (C+D) Preoperative AP and lateral X-ray views after removal of the external fixator, demonstrating the persistent femoral defect before definitive reconstruction. AP: anteroposterior

**Figure 5:**
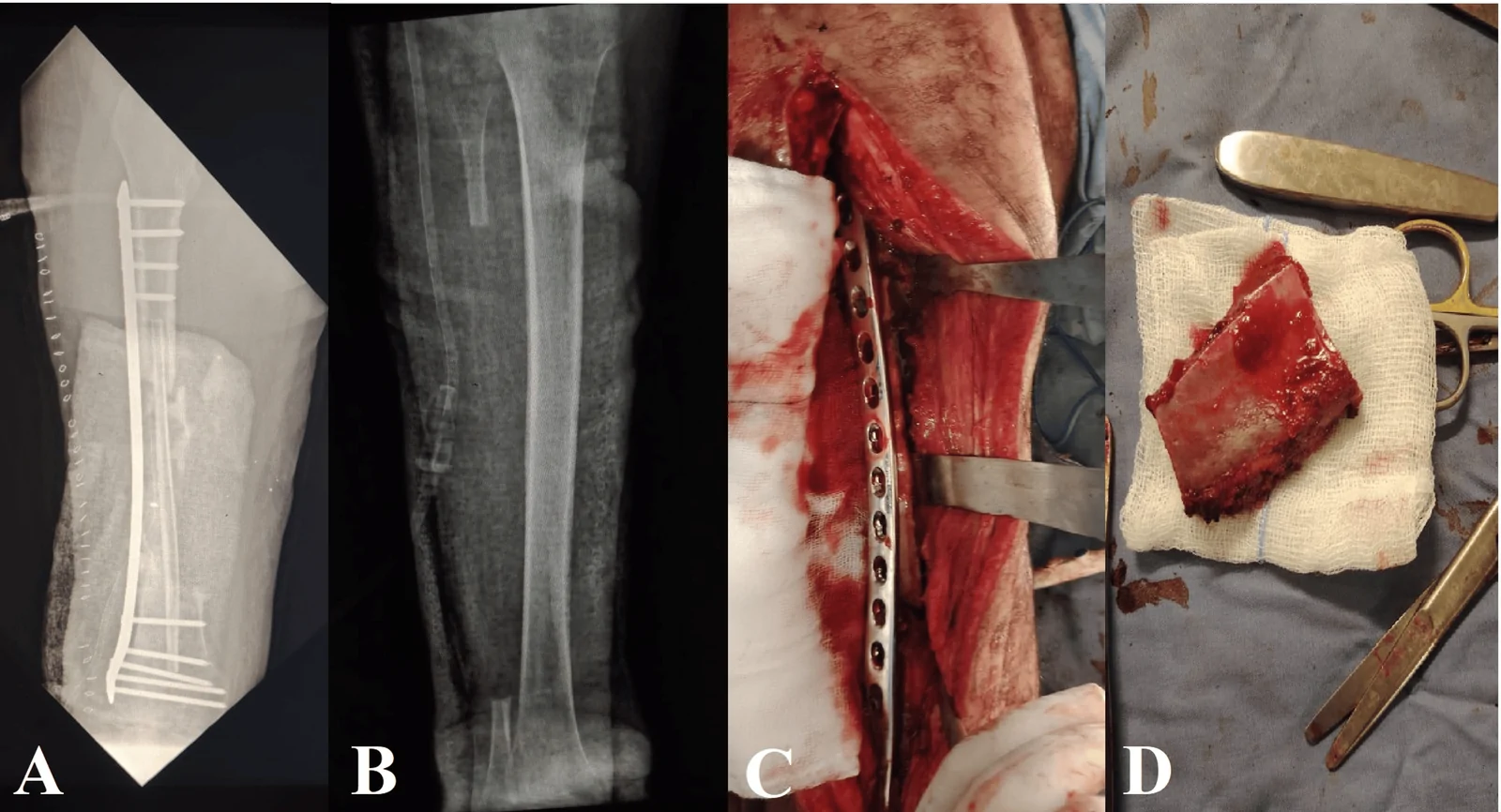
Intraoperative gross images and initial postoperative X-rays (A) Initial postoperative AP X-ray view showing the final NVFG positioning and fixation with distal femur LCP, with final length and alignment. (B) Postoperative AP view of the right leg showing the donor site of the NVFG. (C) Intraoperative clinical photo showing the intramedullary application of the NVFG with fixation by a distal femur LCP plate. (D) Intraoperative clinical photo showing the iliac crest graft after harvesting. AP: anteroposterior; NVFG: non-vascularized fibular graft; LCP: locking compression plate

Postoperatively, the patient was mobilized with protected weight bearing and underwent regular clinical and radiological follow-up. Progressive healing was observed, with radiological union, defined by bridging callus across at least three cortices, achieved at seven months postoperatively. At the latest follow-up, 18 months after surgery, the patient was fully weight bearing without assistive devices and had regained a knee range of motion of 0°-120°. No infection, implant failure, refracture, or donor-site morbidity was observed (Figure 6).

**Figure 6:**
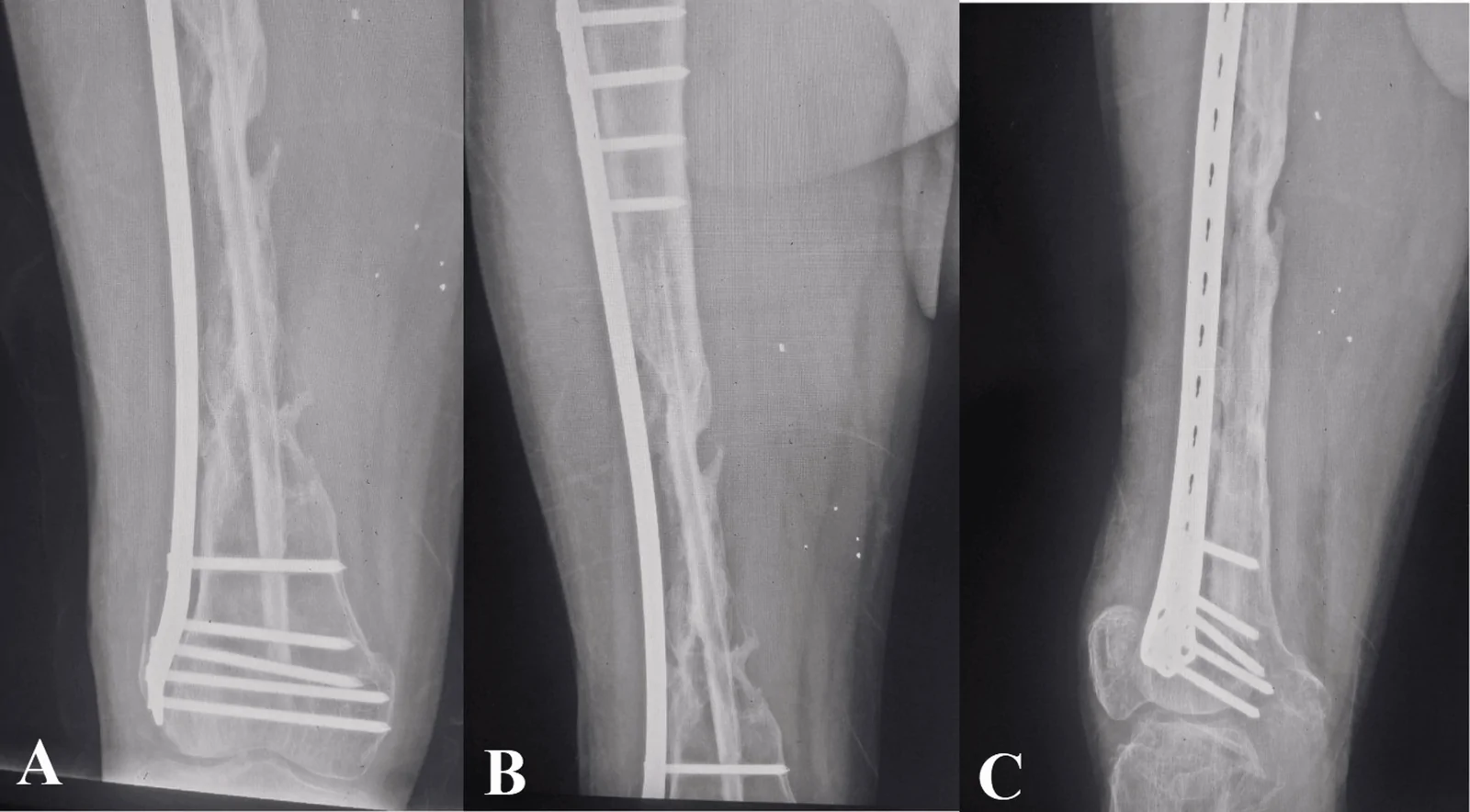
X-rays from the nine-month follow-up Nine-month follow-up AP (A+B) and oblique (C) femur views showing complete union of the fracture site with almost complete femorization of the fibular graft. AP: anteroposterior

Case 3

In Case 3, a 39-year-old male patient sustained a blast injury in April 2024, resulting in a Gustilo-Anderson type IIIC open fracture of the left femur. Initial management included surgical debridement, skeletal traction of the distal femur, and femoral artery bypass. Three months later, he presented with persistent pain, inability to bear weight, limb shortening, and deformity, with no clinical or radiological evidence of fracture healing (Figure 7). The wounds had healed without clinical evidence of infection. Hip range of motion was preserved, while knee examination demonstrated stiffness. Radiographs demonstrated an established femoral nonunion with an 11-cm segmental defect and no significant callus formation. Laboratory investigations showed a complete blood count within normal limits, an erythrocyte sedimentation rate of approximately 30 mm/hour, and negative C-reactive protein, with infection excluded clinically and by laboratory testing. Following thorough debridement and resection of nonviable bone until viable bleeding bone was reached, single-stage reconstruction of the 11-cm defect was performed using an ipsilateral NVFG combined with autologous ICBG, with stabilization using a proximal femoral LCP (Figure 8). Manipulation under anesthesia was performed during the same procedure to address knee stiffness, achieving 90° of knee flexion intraoperatively. No intraoperative complications occurred. Postoperatively, the patient was mobilized with protected weight bearing and underwent regular clinical and radiological follow-up. Progressive healing was observed, with radiological union, defined by bridging callus across at least three cortices, achieved at six months. At the final follow-up, 18 months after surgery, complete fracture healing was confirmed, and the patient was fully weight bearing without assistive devices, with a knee range of motion of 0°-90°. No infection, implant failure, refracture, or donor-site morbidity was observed (Figure 9).

**Figure 7:**
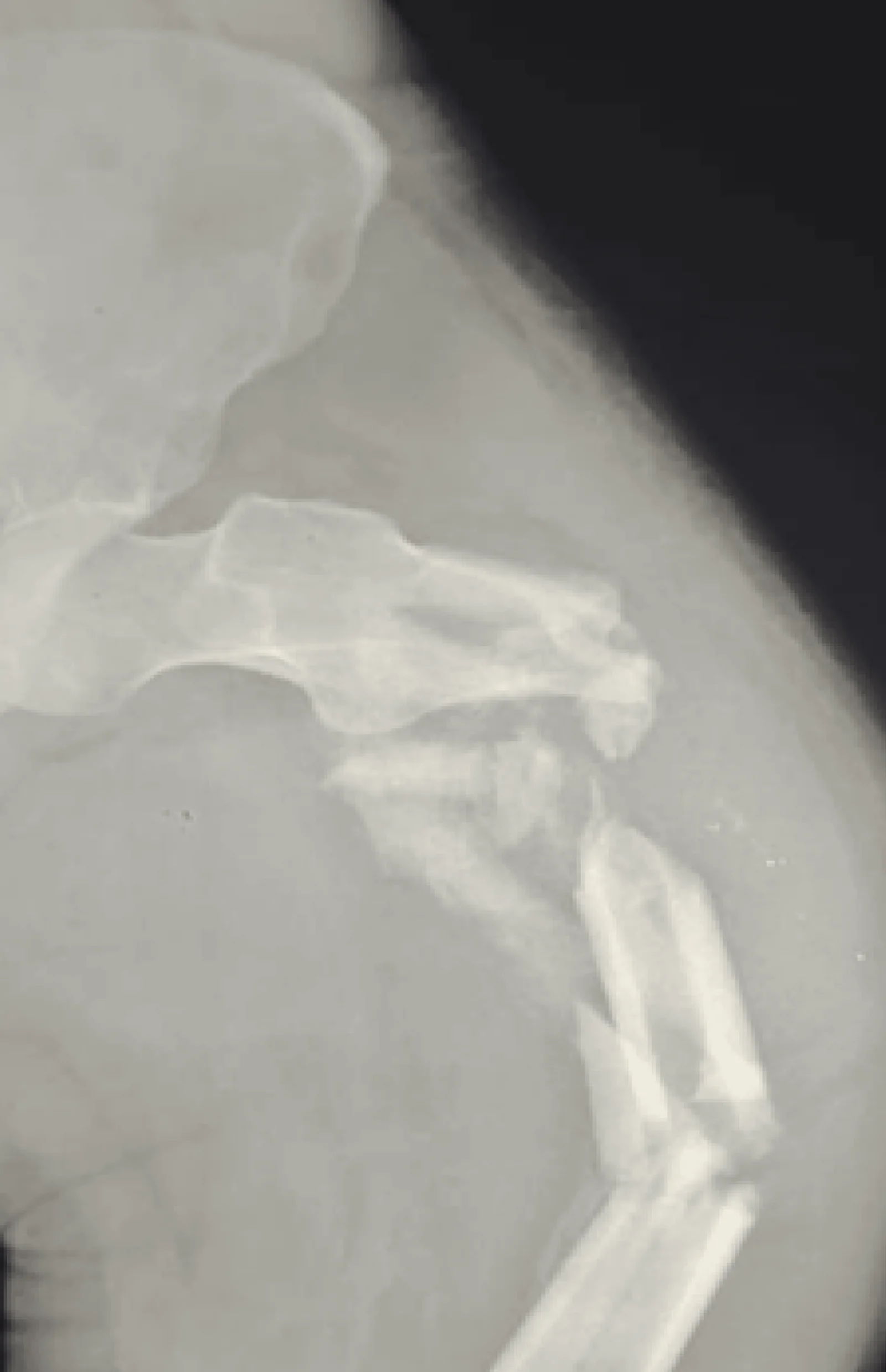
X-rays from the initial presentation Initial presentation of the left femur in AP view showing femur deformity and segmental femur nonunion with bone loss. AP: anteroposterior

**Figure 8:**
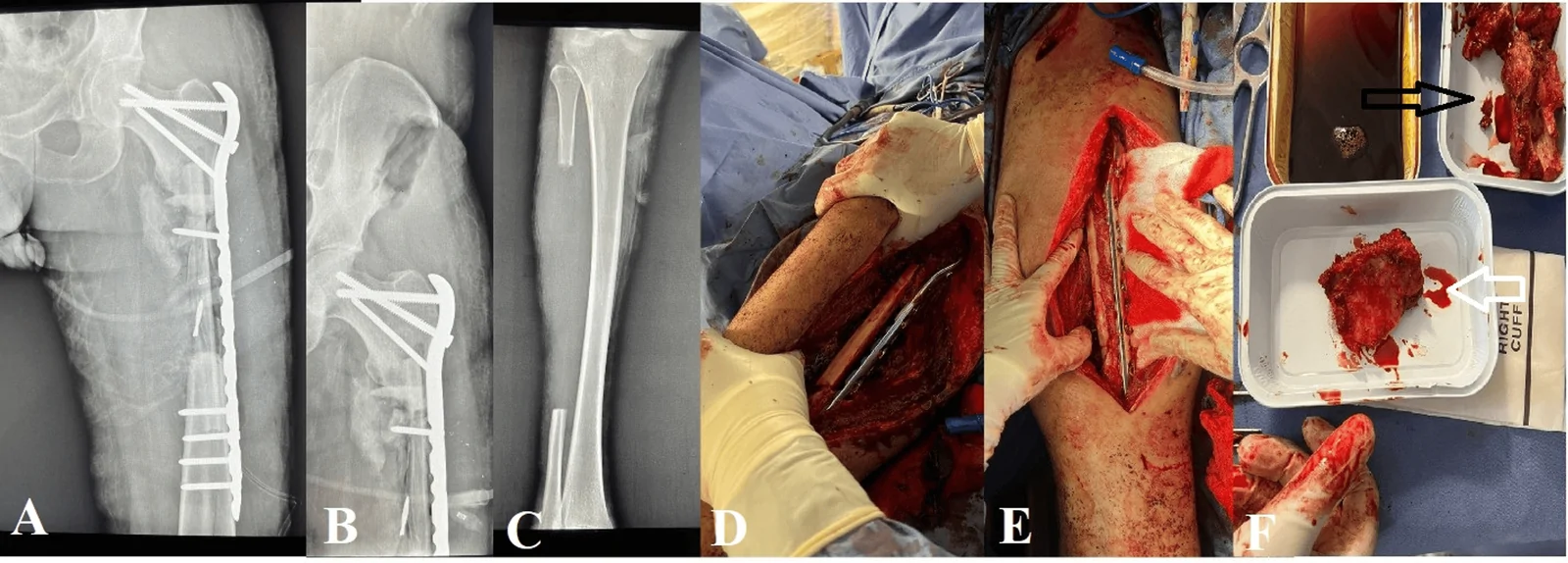
Intraoperative gross images and initial postoperative X-rays (A) Initial postoperative AP X-ray view showing the final alignment and position of the NVFG and the fracture fixation with proximal femur LCP. (B) Left iliac wing X-ray showing the donor site of the iliac crest graft. (C) Postoperative AP view of the left leg showing the donor site of the NVFG. (D) Intraoperative clinical photo showing the final position of intramedullary application of the NVFG with fixation by a proximal femur LCP plate. (E) Intraoperative clinical photo showing the application of the iliac crest blocks between the NVFG and the plate. (F) Intraoperative clinical photo showing the iliac crest graft (white arrow) and the debrided dead bone (black arrow). AP: anteroposterior; NVFG: non-vascularized fibular graft; LCP: locking compression plate

**Figure 9:**
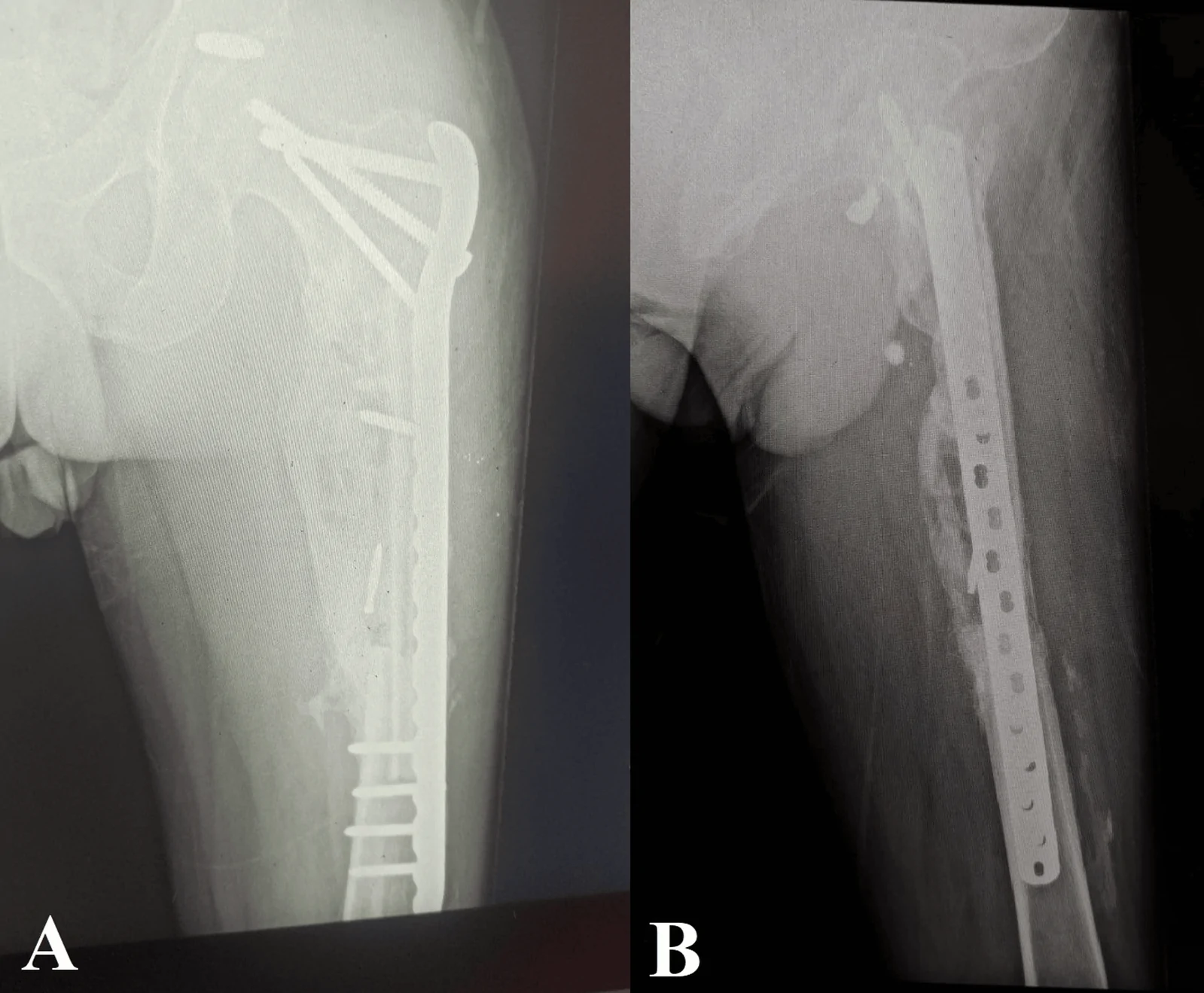
Nine-month follow-up X-rays Nine-month follow-up in AP view (A) and lateral view (B) of the left femur showing complete fracture union. AP: anteroposterior

Case 4

Case 4 describes a 12-year-old boy who sustained a blast injury in January 2024, resulting in a Gustilo-Anderson type IIIC open fracture of the left femur. Initial management included surgical debridement, femoral artery bypass, and fracture stabilization using an external fixator. His treatment subsequently required multiple additional debridement procedures and soft-tissue coverage with skin grafting, followed by definitive fracture fixation three months after the initial injury.

Four months after definitive fixation, he presented to the Jordanian Field Hospital with persistent left thigh pain and inability to bear weight (Figures 10-11). Clinical evaluation demonstrated a healed soft-tissue envelope without signs of active infection, while radiographic assessment revealed implant failure associated with femoral nonunion and a resulting 7-cm bone defect. Laboratory investigations demonstrated a complete blood count within normal limits, an erythrocyte sedimentation rate of approximately 30 mm/hour, and negative C-reactive protein, with infection excluded both clinically and and by laboratory testing. Following thorough debridement and resection of nonviable bone until viable bleeding bone was reached, single-stage reconstruction was performed using an ipsilateral NVFG combined with autologous ICBG, stabilized using an LCP (Figure 12). No intraoperative complications occurred.

**Figure 10:**
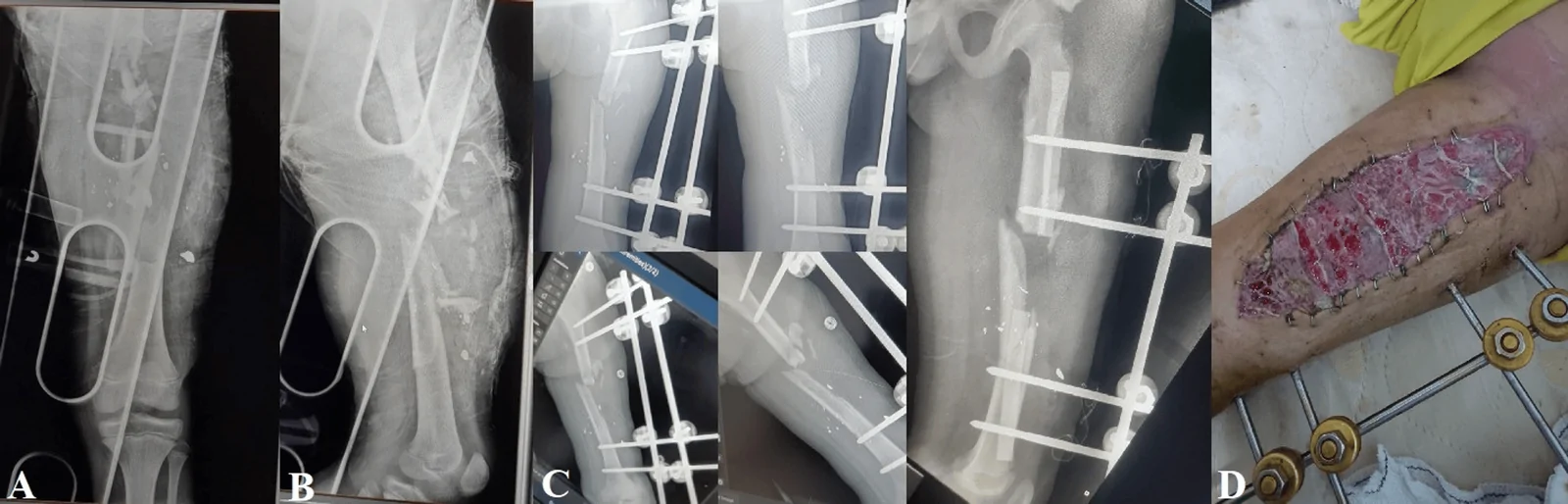
Initial injury X-rays and gross images Initial injury (January 2024) AP (A) and lateral (B) left femur X-ray views showing a left segmental femur open fracture with bone loss and multiple shrapnel fragments. (C) Multiple femur X-ray views showing temporary fracture fixation with an external fixator and bone loss. (D) Clinical gross photo of the thigh showing skin graft application to cover the soft tissue injury. AP: anteroposterior

**Figure 11:**
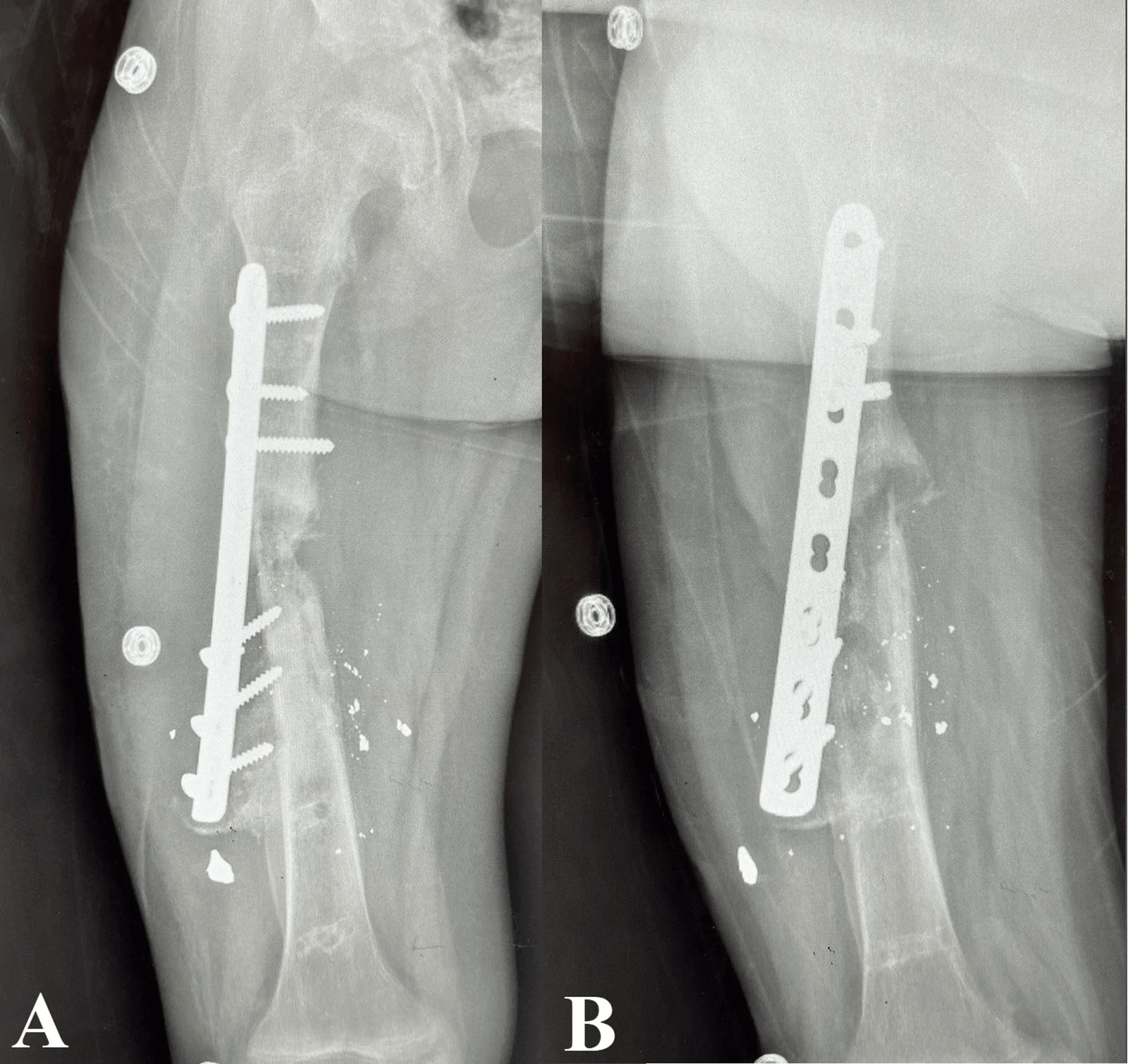
Initial presentation X-rays Initial presentation of AP (A) and lateral (B) views of the left femur showing persistent femoral nonunion with metal failure. AP: anteroposterior

**Figure 12:**
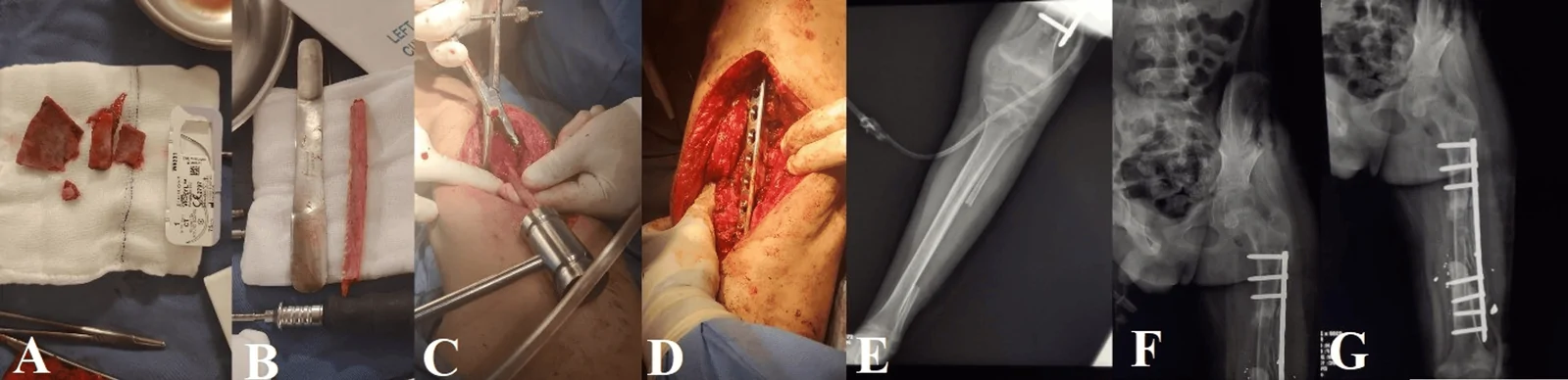
Intraoperative gross images and initial postoperative X-rays (A) Gross intraoperative photo showing the iliac crest graft blocks. (B) Intraoperative clinical photo showing the NVFG final shape and length pre-intramedullary application. (C) Gross intraoperative photo showing the intramedullary application procedure of the NVFG. (D) Gross intraoperative photo showing the final NVFG positioning and the fixation of the femur by straight LCP. (E) Postoperative AP view of the left leg showing the donor site of the NVFG. (F) Left iliac wing X-ray showing the donor site of the iliac crest graft. (G) Initial postoperative AP X-ray view showing the final length and alignment of the femur fracture and the NVFG. AP: anteroposterior; NVFG: non-vascularized fibular graft; LCP: locking compression plate

Postoperatively, the patient was mobilized with protected weight bearing and underwent regular clinical and radiological follow-up. Progressive healing was observed, with radiological union, defined by bridging callus across at least three cortices, achieved at six months postoperatively. At the latest follow-up, 18 months after surgery, complete fracture healing was maintained, and the patient was fully weight bearing without assistive devices, with a knee range of motion of 0°-130°. No recurrent infection, implant failure, refracture, or donor-site morbidity was observed. However, a residual limb-length discrepancy of approximately 4 cm remained (Figure 13).

The distal femoral physis remained open at the latest follow-up, with no evidence of direct physeal injury or growth arrest, as the original fracture and reconstruction were confined to the femoral diaphysis. Although further femoral growth is expected, this was not anticipated to correct the established approximately 4-cm limb-length discrepancy. The patient and family were counseled regarding the expected course and the potential option of elective limb-lengthening when appropriate medical and surgical resources become available. In the interim, he uses a medically prescribed shoe lift, with satisfactory functional tolerance of the residual discrepancy.

**Figure 13:**
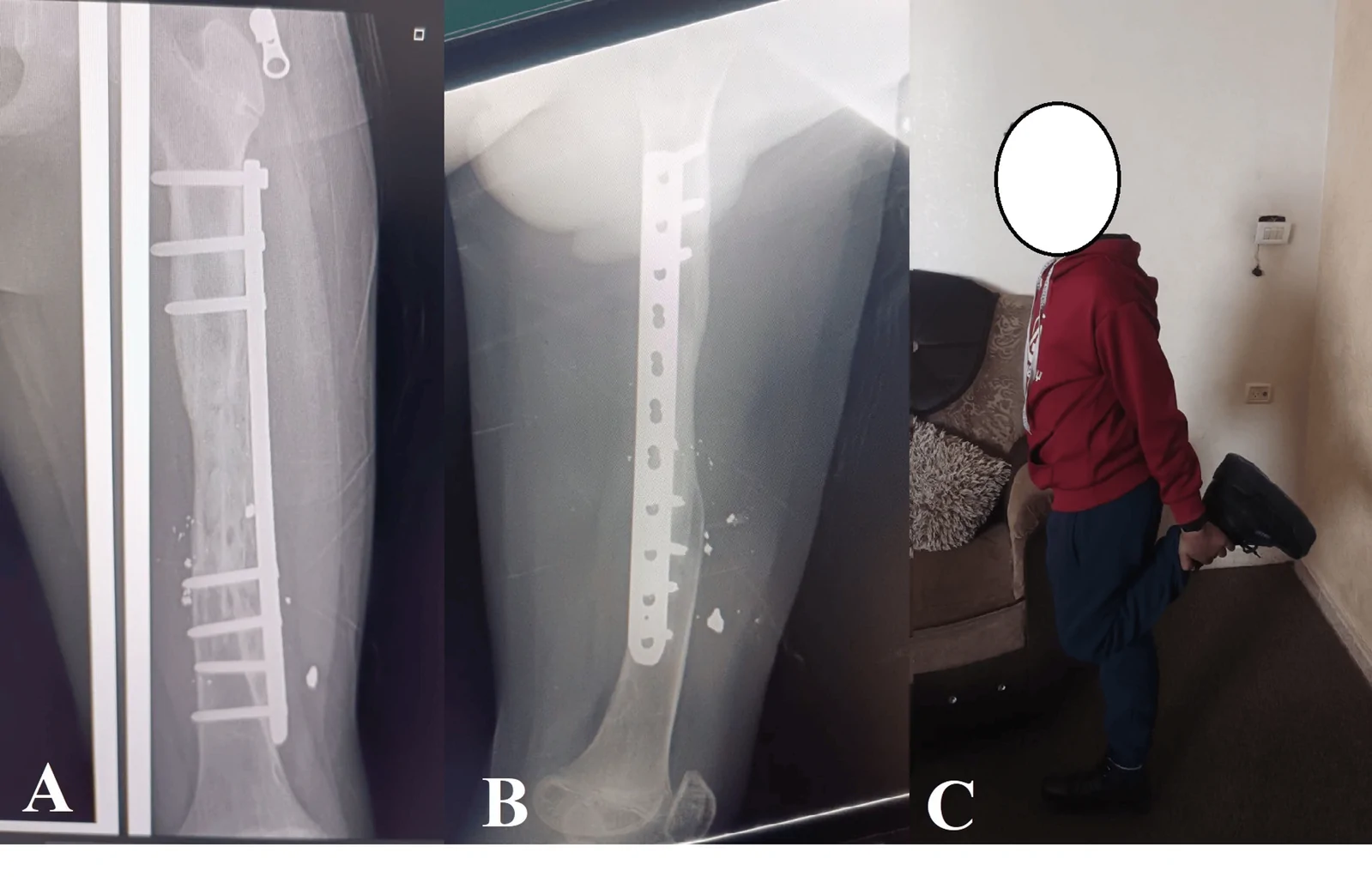
Nine-month follow-up X-rays and gross images Nine-month follow-up AP (A) and lateral (B) left femur X-ray views showing complete consolidation and healing of the femur fracture and complete femorization of the NVFG. (C) Clinical photograph showing the final full knee flexion of the same limb. AP: anteroposterior; NVFG: non-vascularized fibular graft

Case 5

Case 5 describes a 40-year-old woman who sustained a blast injury in March 2024, resulting in a closed fracture of the left femur. Initial management consisted of fracture stabilization using an external fixator. Six months later, she presented to the Jordanian Field Hospital with persistent symptoms and no clinical or radiological evidence of fracture healing. Clinical examination demonstrated persistent functional impairment, while the soft-tissue condition was satisfactory without clinical evidence of infection. Radiographs confirmed an established nonunion without significant progression of healing. Laboratory investigations, including complete blood count, erythrocyte sedimentation rate, and C-reactive protein, showed no evidence of active infection. Following exclusion of infection, definitive reconstruction was planned. Intraoperatively, extensive devascularization and nonviability of the femoral bone were encountered, with no bleeding from the involved bone ends. Thorough debridement and resection were therefore continued until viable bleeding bone was reached, resulting in a 24-cm segmental defect. Given the exceptionally large defect and the shorter length of the available fibular graft, approximately 2-3 cm of limb shortening was accepted to facilitate adequate graft seating, maximize host-graft contact, and improve mechanical stability. Reconstruction was performed using a 27-cm NVFG combined with autologous ICBG, stabilized with a long distal femoral LCP (Figure 14). No intraoperative complications occurred.

**Figure 14:**
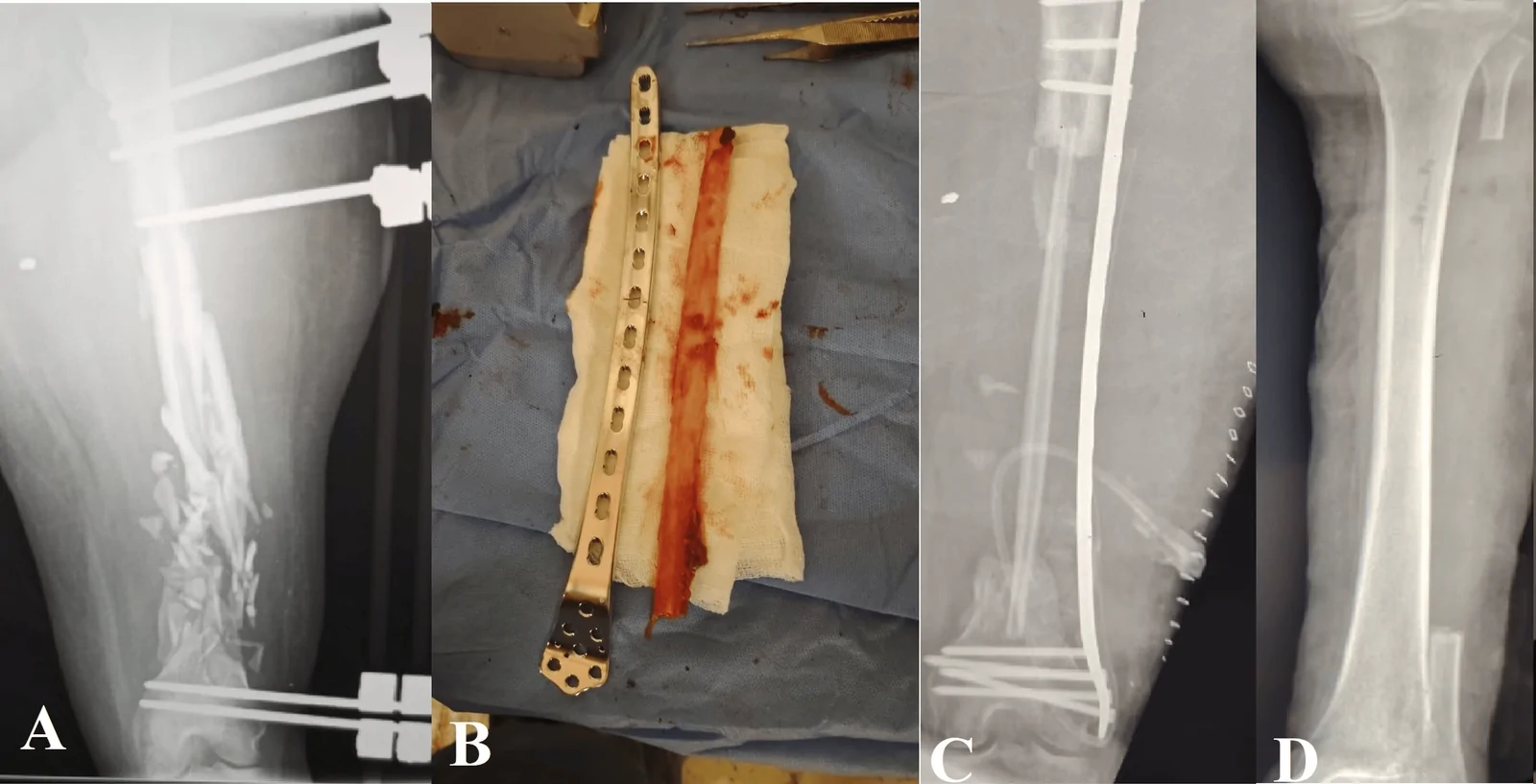
Initial presentation, intraoperative gross images and initial postoperative X-rays (A) Initial presentation left femur AP X-ray view showing segmental comminuted femur fracture nonunion with a gap. (B) Clinical intraoperative gross photo showing the length of the NVFG in comparison with the 13-hole distal femur LCP from Synthes®. (C) Initial postoperative femur AP X-ray view showing the final length and alignment of the left femur with fixation using a distal femur LCP. (D) Left leg AP view showing the donor site of the NVFG and the remaining proximal and distal parts of the fibula. AP: anteroposterior; NVFG: non-vascularized fibular graft; LCP: locking compression plate

The patient was mobilized with protected weight bearing and followed clinically and radiologically for 18 months postoperatively. Progressive healing and stable integration of the reconstruction were observed, resulting in a stable functional union despite persistence of a residual medial bone gap measuring approximately 2-4 cm in the distal third of the femur. At the latest follow-up after 18 months postoperatively, radiographs demonstrated bridging and consolidation across three of the four cortices, with a residual approximately 4-cm unbridged area along the distal medial aspect of the graft. Given the substantial radiographic healing and the patient's clinical status, the outcome was considered functional union/healing rather than complete radiographic union. She was able to ambulate with full weight bearing without assistive devices, pain, or significant functional limitation. Knee range of motion was from -10° of hyperextension to 100° of flexion. No infection, implant failure, refracture, or donor-site morbidity was observed (Figure 15).

**Figure 15:**
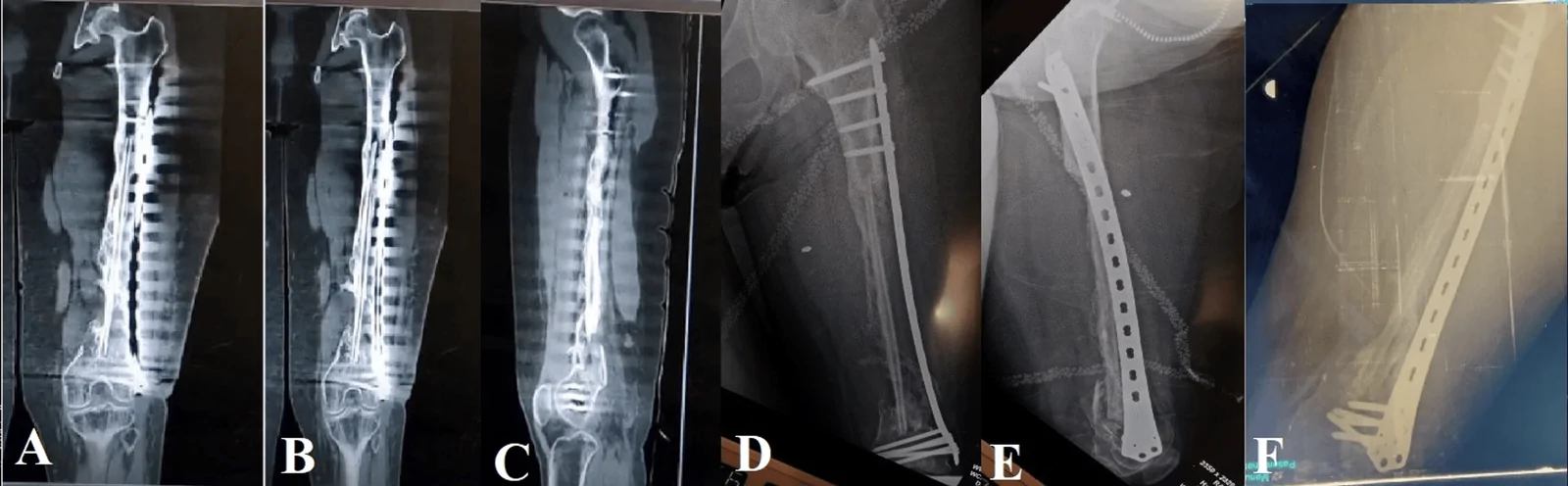
Follow-up CT-scan images and X-rays Follow-up CT scan images and X-rays. (A+B) Twelve-month coronal CT images showing fracture healing. (C) Twelve-month sagittal CT image. (D) Fourteen-month follow-up AP X-ray view, (E) lateral X-ray view, and (F) oblique X-ray view showing healing of the femoral nonunion with callus formation. AP: anteroposterior

Case 6

Case 6 involved a 39-year-old man who sustained a blast injury in December 2023, resulting in a Gustilo-Anderson type IIIC open fracture of the right femur. Initial management included thorough surgical debridement, femoral artery bypass, and fracture stabilization using an external fixator.

Eight months later, he presented to the Jordanian Field Hospital with persistent pain, inability to bear weight, and an established femoral nonunion. The wounds had healed without clinical evidence of active infection. Clinical examination demonstrated preserved hip motion with associated knee stiffness. Radiographs confirmed femoral nonunion with a segmental bone defect and no significant callus formation. Laboratory investigations showed a complete blood count within normal limits, an erythrocyte sedimentation rate of approximately 30 mm/hour, and negative C-reactive protein, with no clinical or laboratory evidence of active infection. Following exclusion of infection, thorough debridement and resection of nonviable bone until viable bleeding bone was reached resulted in a 15-cm segmental defect. Single-stage reconstruction was performed using an ipsilateral NVFG combined with autologous ICBG, with stabilization achieved using a straight LCP (Figure 16). No intraoperative complications occurred.

**Figure 16:**
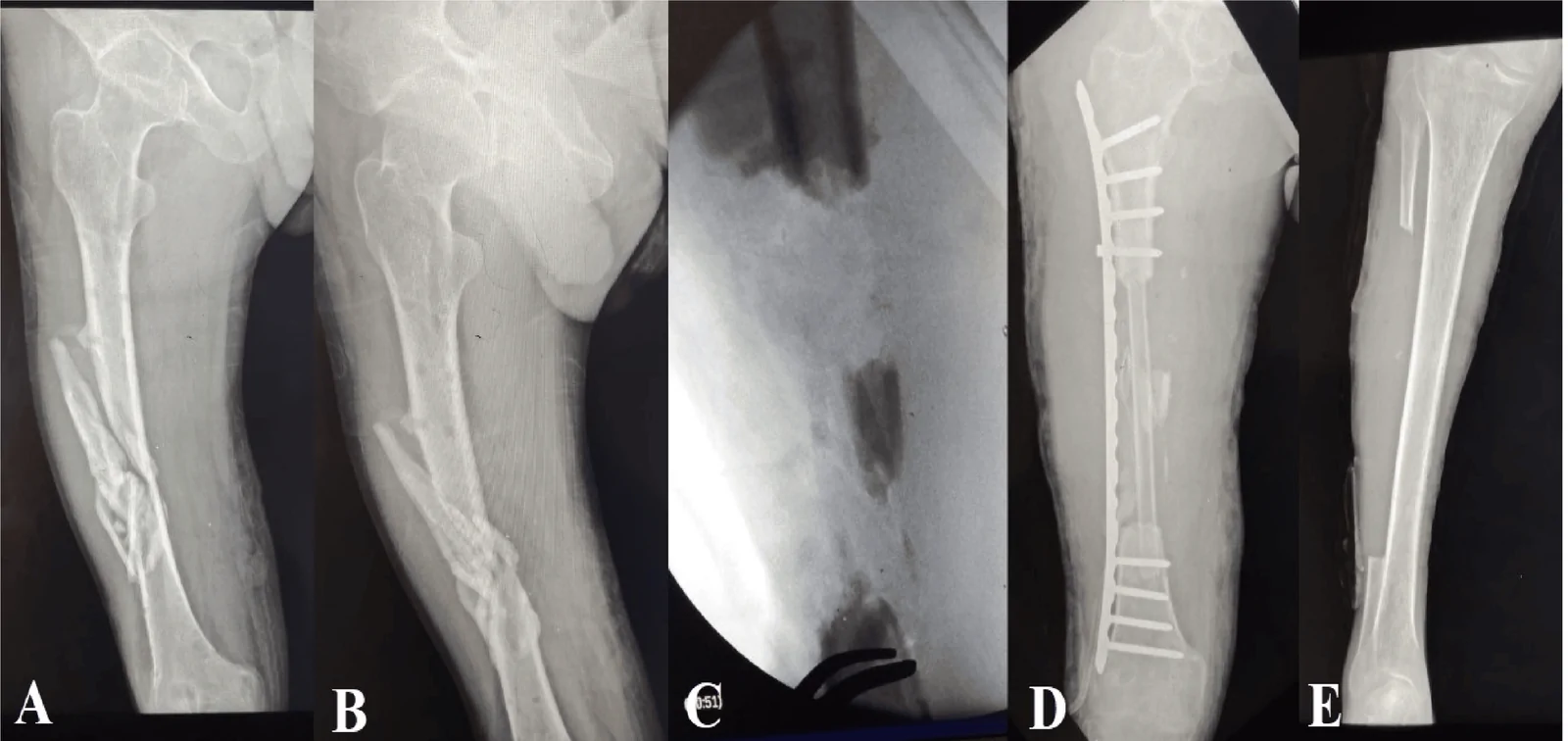
Initial presentation, intraoperative fluoroscopic and initial postoperative X-rays (A+B) Initial preoperative AP and oblique right femur X-ray views showing femur nonunion. (C) Intraoperative fluoroscopic image showing the final gap length after excision and debridement of the necrotic and dead bone. (D) Initial postoperative AP X-ray view showing the final length and alignment of the right femur after fixation with a straight LCP and application of the NVFG and iliac crest graft. (E) Postoperative right leg AP X-ray view showing the NVFG donor site. AP: anteroposterior; LCP: locking compression plate; NVFG: non-vascularized fibular graft

Postoperatively, the patient was mobilized with protected weight bearing and underwent regular clinical and radiological follow-up. Progressive healing was observed, with radiological union, defined by bridging callus across at least three cortices, achieved at eight months post-reconstruction. At the final follow-up, 18 months after surgery, complete fracture healing was maintained, and the patient was fully weight bearing without assistive devices. Knee range of motion was 0°-100°, with no evidence of infection, implant failure, refracture, or donor-site morbidity (Figure 17).

**Figure 17:**
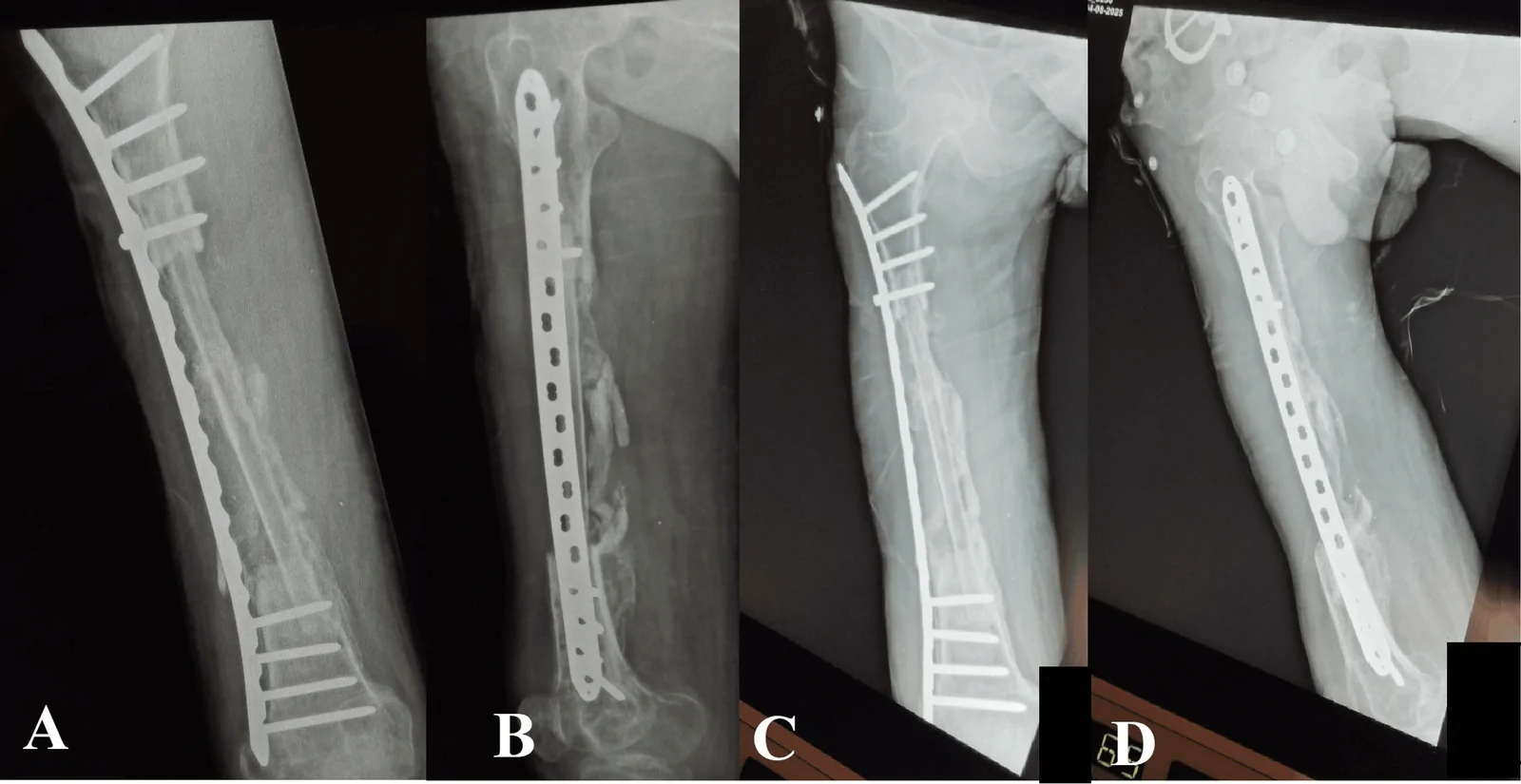
Follow-up X-rays (A+B) Three-month follow-up AP and lateral X-ray views showing progressive healing and callus formation. (C+D) Eight-month follow-up AP and lateral X-ray views showing complete femoral fracture healing. AP: anteroposterior

Case 7

In Case 7, a 33-year-old man sustained a gunshot injury in November 2023, resulting in a Gustilo-Anderson type IIIA open fracture of the right proximal femur. Initial management included thorough surgical debridement followed by open reduction and internal fixation using a proximal femoral LCP.

Ten months after the initial injury, he presented to the Jordanian Field Hospital with persistent pain during weight bearing and functional limitation of the affected limb. The soft-tissue wounds had healed, with no clinical evidence of active infection. Radiographs demonstrated an established proximal femoral nonunion (Figure 18). Laboratory investigations showed a complete blood count within normal limits, an erythrocyte sedimentation rate of approximately 30 mm/hour, and negative C-reactive protein, with no clinical or laboratory evidence of active infection. Following exclusion of infection, revision surgery was performed. The previous implant was removed, and thorough debridement with excision of necrotic and nonviable bone was carried out until viable bleeding bone was reached, resulting in a 6-cm segmental defect. Single-stage reconstruction was then performed using a NVFG combined with autologous ICBG, with stabilization achieved using a dynamic condylar screw (DCS). No intraoperative complications occurred.

**Figure 18:**
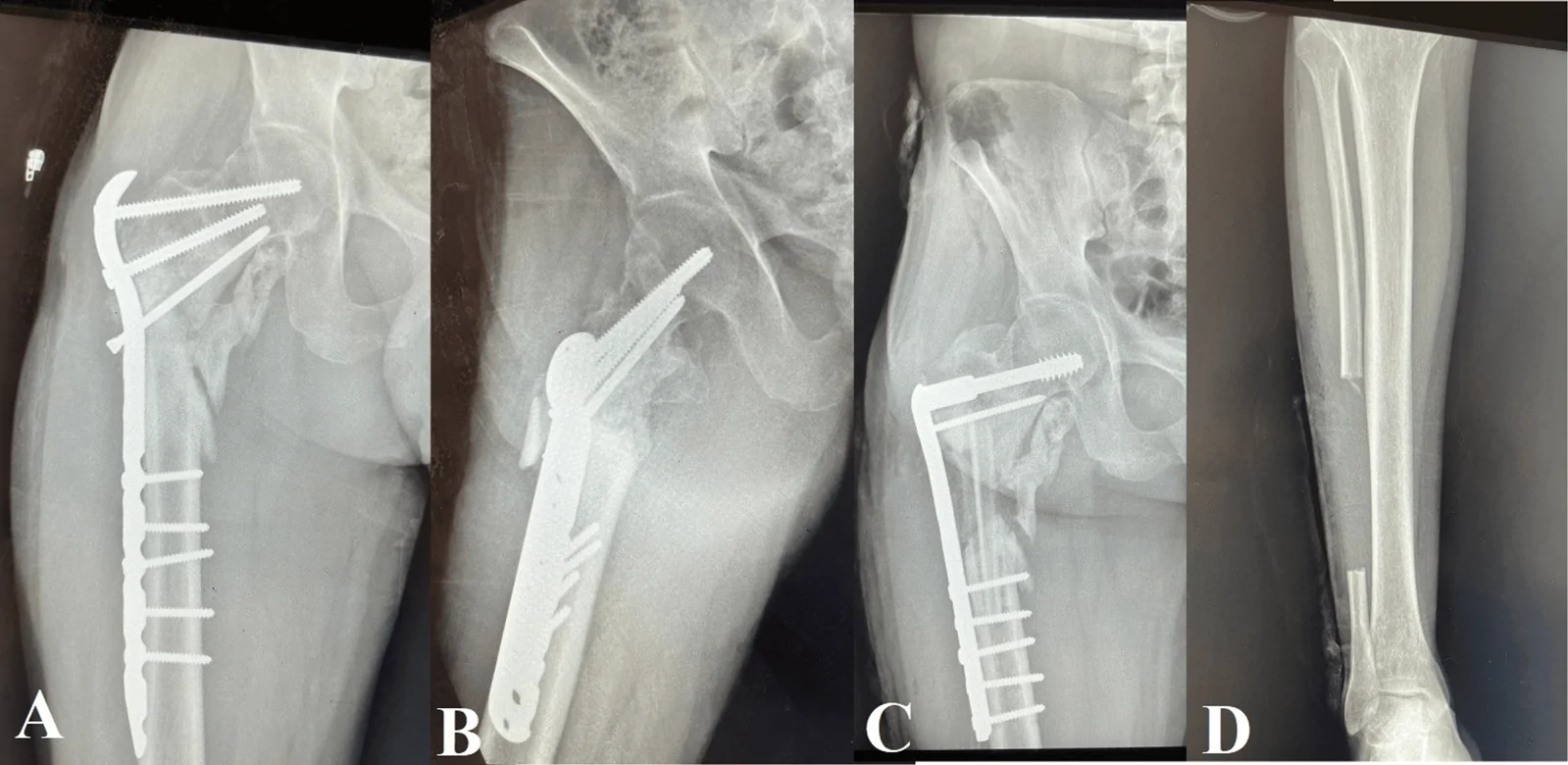
Initial presentation and initial postoperative X-rays Initial presentation AP (A) and lateral (B) right femur X-ray views showing well-established right proximal femur nonunion. (C) Initial postoperative AP right hip view showing the NVFG and iliac crest graft in final position and right femur alignment after fixation with a DCS plate. (D) Postoperative right leg X-ray AP view showing the NVFG donor site. AP: anteroposterior; NVFG: non-vascularized fibular graft; DCS: dynamic condylar screw

Postoperatively, the patient was mobilized with protected weight bearing and underwent regular clinical and radiological follow-up. Progressive healing was observed, and clinical and radiological union, defined by bridging callus across at least three cortices, was achieved at nine months postoperatively. At the final follow-up, 18 months after surgery, complete fracture healing was maintained. The patient was ambulating independently with full weight bearing and without assistive devices, demonstrating satisfactory restoration of hip and knee function, with knee range of motion from 0° to 130°. No recurrent infection, implant failure, refracture, or donor-site morbidity was observed (Figure 19). 

**Figure 19:**
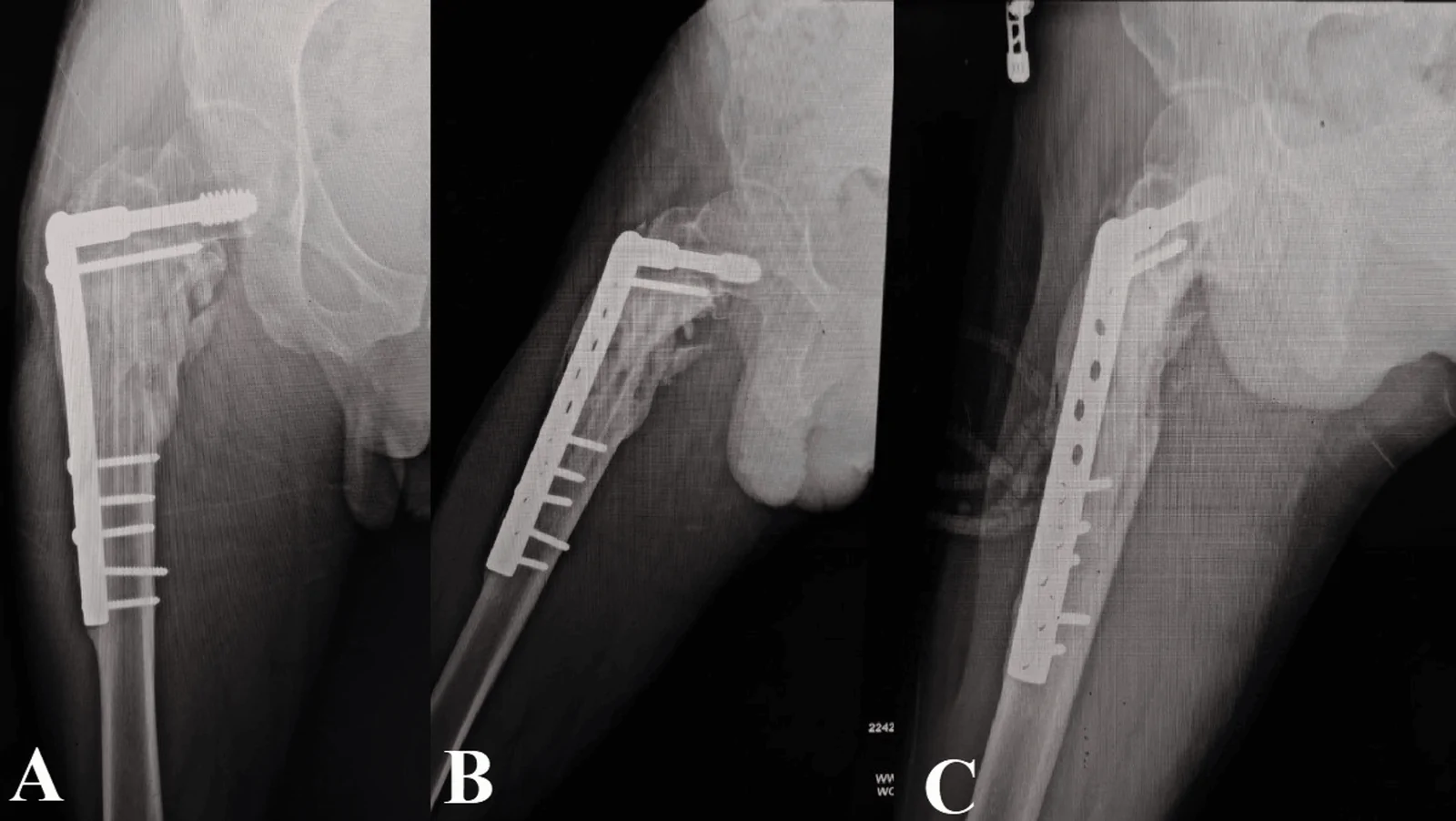
Follow-up X-rays Fourteen-month follow-up right hip AP (A), oblique (B), and lateral (C) X-ray views showing complete fracture healing and good callus formation. AP: anteroposterior

Case 8

In Case 8, a 14-year-old girl sustained a blast-related Gustilo-Anderson type IIIA proximal femoral fracture approximately 10 months before presentation to our team. She initially underwent fixation with a dynamic condylar screw at the time of injury. On presentation to the Jordanian Field Hospital in early August 2024, she had an established infected nonunion with an active draining sinus. Laboratory investigations demonstrated an elevated erythrocyte sedimentation rate (ESR) of 88 mm/hour and a positive C-reactive protein (CRP), supporting the diagnosis of an active infected nonunion. A first-stage procedure was therefore performed, consisting of implant removal, extensive debridement, intraoperative microbiological sampling, and fixation with a straight LCP combined with an antibiotic-impregnated cement spacer (Figure 20). Initial deep cultures grew *Escherichia coli*. She subsequently received approximately six weeks of intravenous culture-directed antibiotic therapy with gentamicin and meropenem; the ESR initially increased to 150 mm/hour. During this period, she was monitored clinically and with weekly ESR and CRP measurements. The draining sinus and soft-tissue infection progressively resolved, with complete wound healing and no further discharge, while the inflammatory markers progressively normalized. Definitive reconstruction was planned after clinical resolution of the infection. At the time of the second-stage procedure in mid-September 2024, there were no gross intraoperative signs of infection, and three deep tissue samples were obtained for culture, all of which were negative. Based on the clinical resolution of the infection, satisfactory laboratory response, absence of intraoperative evidence of active infection, and negative deep cultures, the cement spacer was removed, and the approximately 6-cm segmental defect was reconstructed using a non-vascularized fibular strut graft combined with autologous ICBG (Figures 21-22).

**Figure 20:**
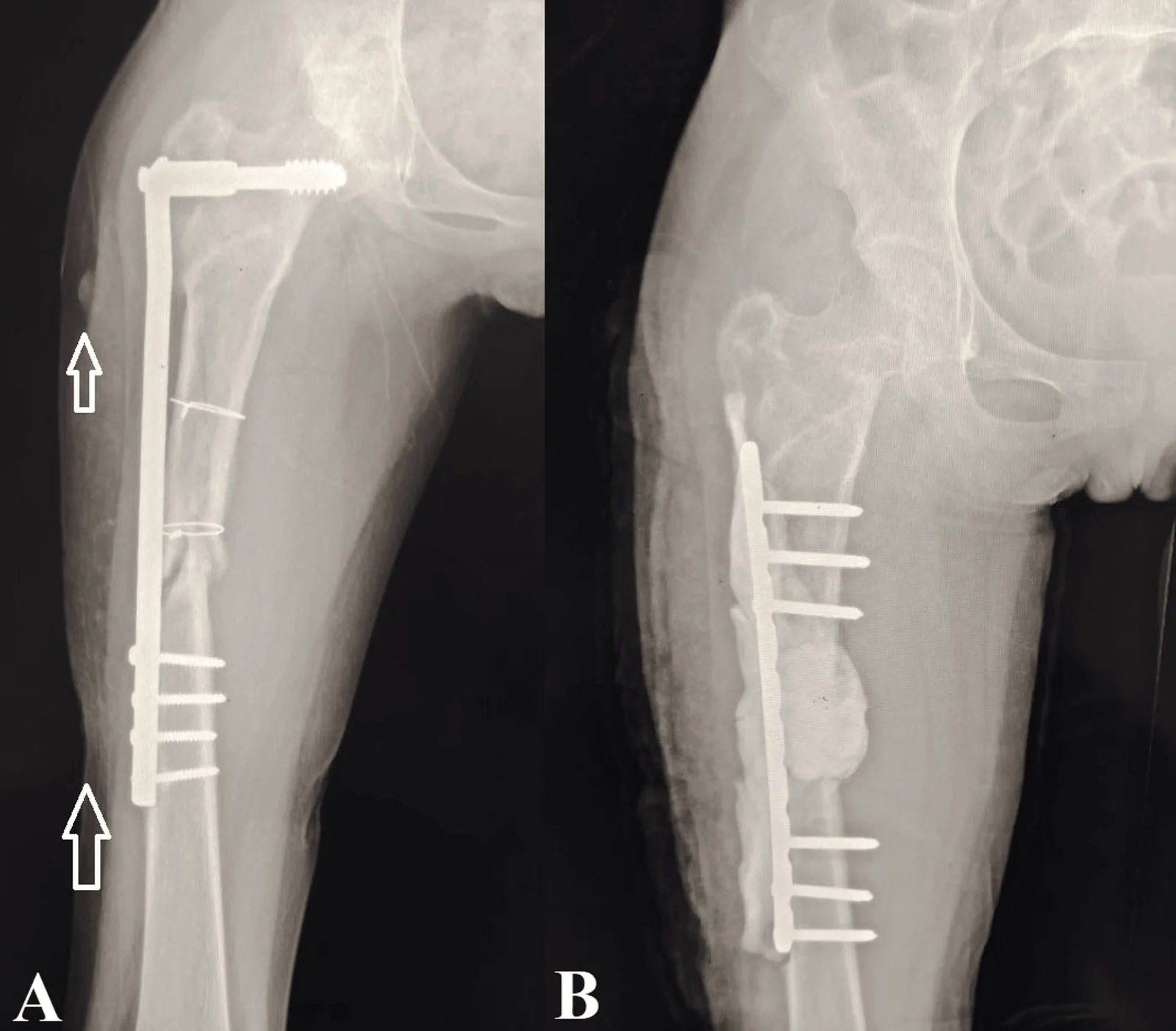
Initial presentation and post-first-stage X-rays (A) Initial presentation right hip and proximal femur X-ray AP view showing proximal femur fracture nonunion, which was fixed with a DCS plate and two cerclage wires, with two sinuses (white arrows) showing infected femur nonunion. (B) First-stage postoperative hip AP view showing DCS plate removal, dead bone excision, and cement spacer application fixed with a straight LCP plate. AP: anteroposterior; DCS: dynamic condylar screw; LCP: locking compression plate

**Figure 21:**
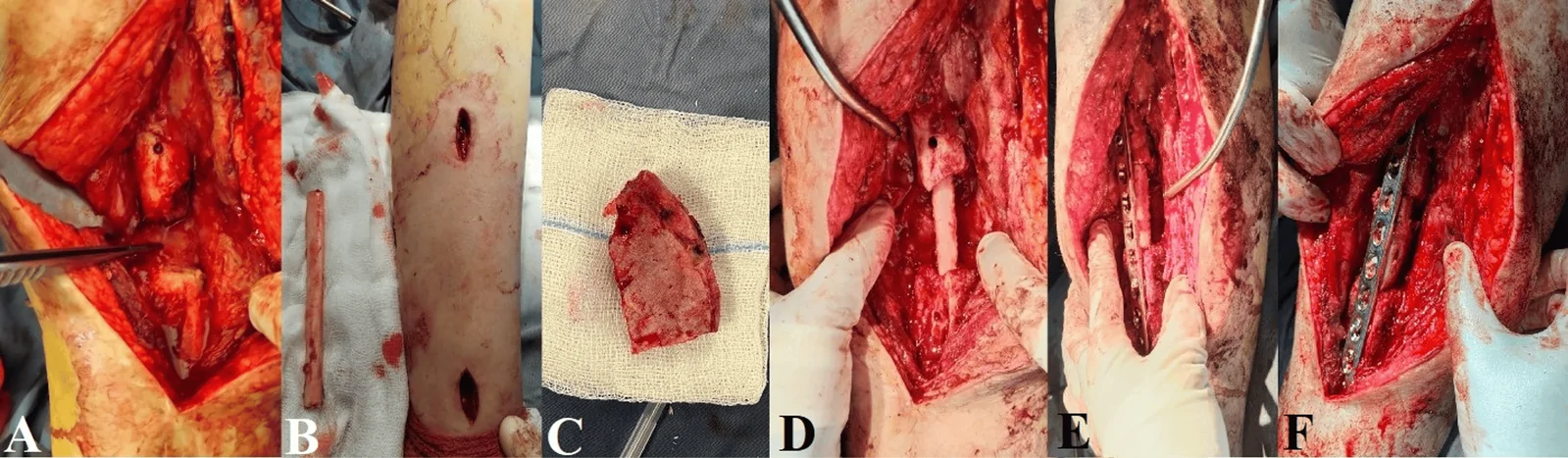
Second-stage intraoperative gross clinical photos (A) Clinical photo showing the pseudomembrane after removal of the cement spacer. (B) Clinical photo showing percutaneous NVFG harvesting. (C) Clinical photo showing the iliac crest graft. (D) Clinical photo showing intramedullary fibular graft application. (E) Clinical photo showing fracture fixation with an LCP straight plate. (F) Clinical photo showing iliac graft block application. NVFG: non-vascularized fibular graft; LCP: locking compression plate

**Figure 22:**
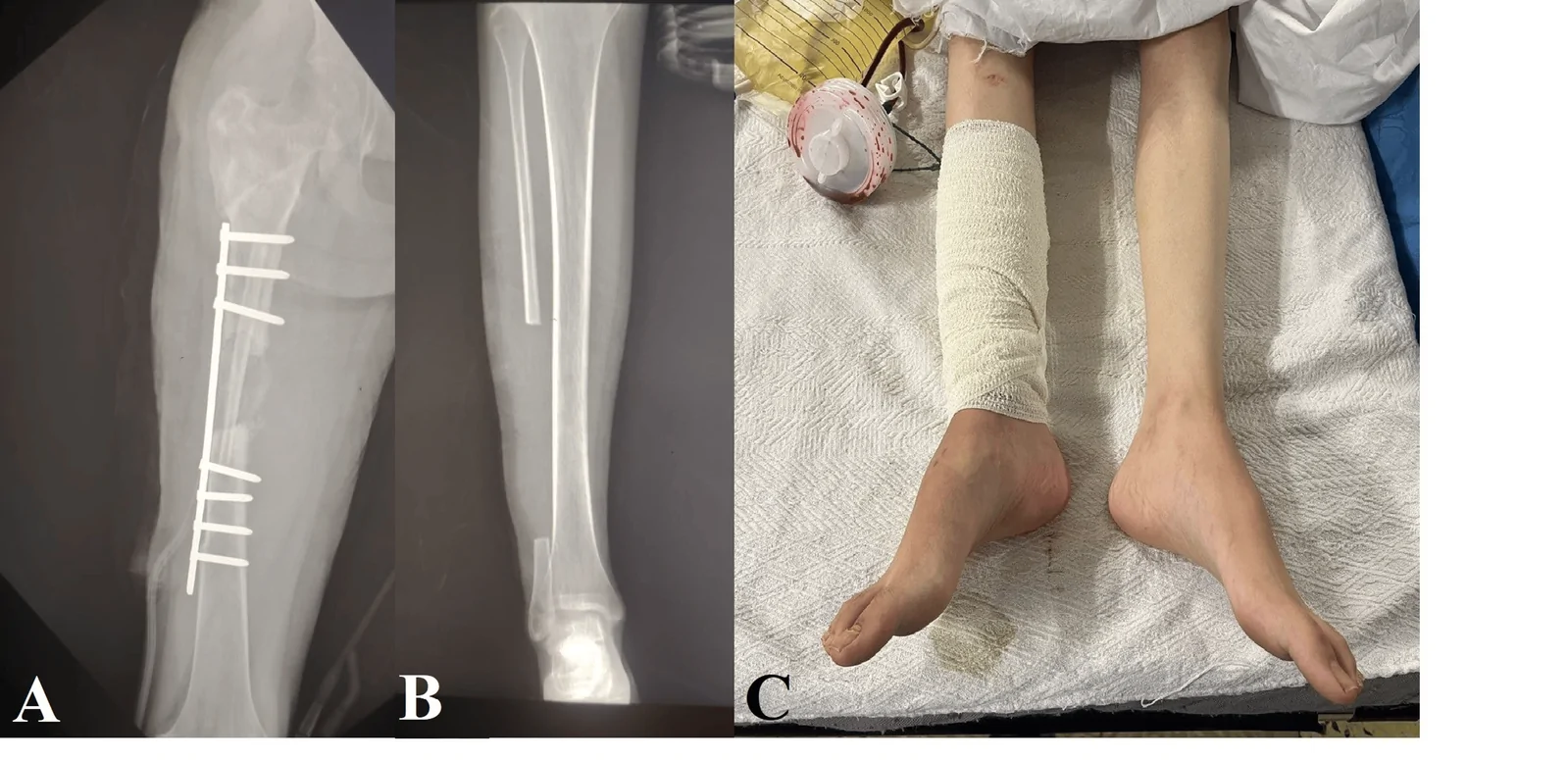
Postoperative gross images and X-rays (A) Initial right femur postoperative X-ray AP view showing the final alignment and length of the right femur after NVFG application and fixation with a straight LCP. (B) Right leg AP X-ray view showing the donor NVFG site. (C) Gross clinical photo showing the final limb length, rotation, and alignment after the second procedure, showing minimal shortening with good alignment. AP: anteroposterior; NVFG: non-vascularized fibular graft; LCP: locking compression plate

Postoperatively, despite explicit instructions regarding protected weight bearing, the patient engaged in premature weight bearing against medical advice, resulting in bending of the fixation plate. Revision with plate exchange was performed at 14 weeks postoperatively (December 2024) at another hospital, after the end of our field-hospital mission (Figure 23). Nevertheless, progressive osseous healing was subsequently achieved, with final consolidation occurring 10 months after the initial reconstruction. At the latest follow-up, 13 months after reconstruction, she was ambulating independently without assistive devices, with no reported recurrence of infection. This case highlights the importance of staged reconstruction and objective monitoring of infection control using clinical findings and serial inflammatory markers, as well as strict adherence to postoperative weight-bearing restrictions to prevent mechanical failure.

**Figure 23:**
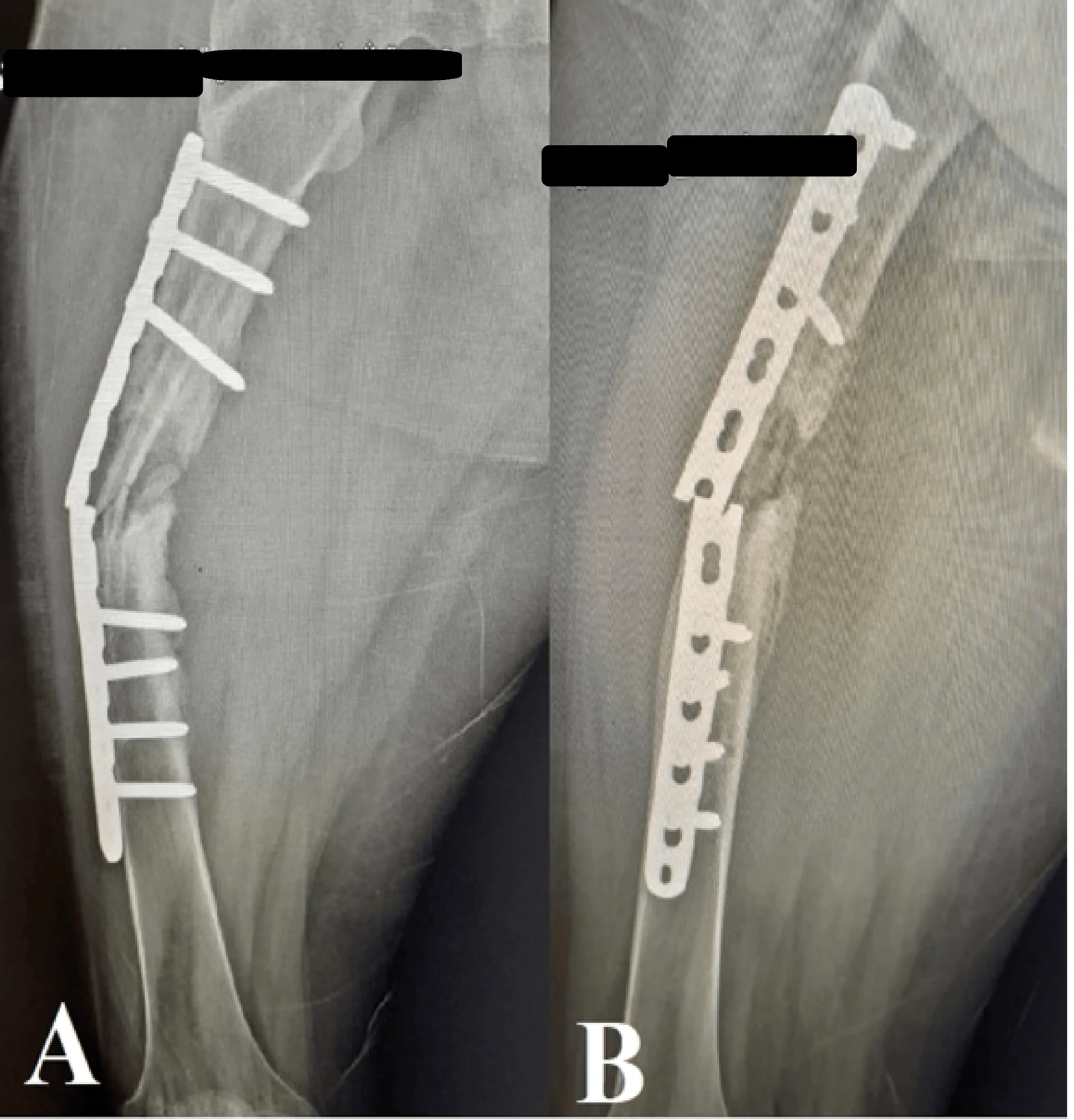
X-rays showing metal failure Fourteen-week follow-up AP (A) and lateral (B) X-ray views showing metal failure with minimal callus formation without signs of infection. AP: anteroposterior

## Discussion

Blast and gunshot injuries are commonly associated with extensive contamination, devitalized tissues, and severe soft-tissue damage, necessitating prompt and repeated debridement to reduce the risks of infection, osteomyelitis, nonunion, and limb loss [12]. In conflict and resource-limited environments such as Gaza, delays in access to definitive care, coupled with disruption of healthcare infrastructure and reliance on short-term visiting surgical teams, often restrict initial management to basic debridement and external fixation. Consequently, many potentially salvageable injuries progress to complex reconstructive problems characterized by infection, stiffness, and established nonunion [5,6].

Management of segmental femoral defects remains a major reconstructive challenge, particularly when defects exceed 5-6 cm or reach a critical size (>2-2.5 times the bone diameter), where spontaneous healing is unlikely, and staged reconstruction is typically required [13]. Cancellous autografting is generally limited to small defects (<4 cm) due to progressive graft resorption in larger gaps [14]. Accordingly, larger defects require more advanced reconstructive strategies, including the Masquelet-induced membrane technique, bone transport, and vascularized or non-vascularized fibular grafting [15]. Although staged protocols involving debridement, infection control, temporary stabilization, and delayed definitive reconstruction have been shown to improve bone healing and graft incorporation, these methods are often technically demanding, resource-intensive, and require specialized expertise. Moreover, robust evidence guiding the optimal reconstructive strategy for very large defects, particularly those exceeding 10 cm, remains limited.

Fibular grafting represents a versatile option in the reconstruction of segmental defects, especially in the presence of infection or compromised biological environments. Vascularized fibular grafts offer superior biological integration and graft viability; however, they are associated with increased operative complexity, longer surgical time, and the need for microsurgical expertise [16]. In contrast, non-vascularized fibular grafts provide a simpler, faster, and more cost-effective alternative, making them particularly suitable in resource-constrained settings. Although a formal economic analysis was not performed, the technique may offer practical resource advantages over reconstructive strategies requiring microsurgical expertise, specialized equipment, or prolonged staged procedures, particularly in conflict-affected and resource-limited settings. Although concerns remain regarding their biological performance in large defects, acceptable union rates have been reported when combined with additional biological augmentation such as cortico-cancellous grafting, with successful outcomes described in defects up to approximately 9 cm [17].

Autologous ICBG remains the gold standard for promoting bone healing in nonunion because of its osteogenic, osteoinductive, and osteoconductive properties. However, its application in large segmental defects is often limited by restricted graft volume and donor-site morbidity, including pain, infection, hematoma, and neurovascular injury, with reported complication rates ranging from 1.8-10% for major complications and 7.1-39% for minor complications [18].

In the present case series, we adopted a pragmatic reconstructive strategy for large segmental femoral defects, including defects exceeding 10 cm, in a conflict setting with severely limited resources. The approach combined NVFG as a structural strut to restore bone continuity and provide mechanical support with ICBG to enhance biological incorporation. A similar combined reconstructive strategy was previously reported by our group for tibial nonunion with segmental bone loss in a war setting, in which all five patients ultimately achieved union despite the complexity of their injuries and the need for revision surgery in two cases [19]. Together, these findings suggest that combining structural NVFG with biological augmentation using autologous ICBG may be a practical reconstructive option for complex segmental bone defects when microsurgical reconstruction or prolonged staged techniques are not readily available. Despite the inherent risks of mechanical failure and other implant-related complications in this high-energy trauma population, satisfactory union was achieved in most cases. These outcomes may have been facilitated by meticulous debridement, appropriate infection control, restoration of limb alignment and length, and stable internal fixation.

This series has several strengths. First, it describes reconstruction of exceptionally large femoral segmental defects, including five defects exceeding 10 cm, a clinical scenario for which experience with non-vascularized fibular grafting remains limited. Second, clinical and radiographic outcomes were assessed from routine postoperative follow-up despite the substantial logistical challenges of providing reconstructive care in an active conflict setting. Third, all procedures were performed by a single surgeon, providing consistency in the operative technique. Finally, the study illustrates pragmatic adaptations of available instrumentation and reconstructive principles to a resource-constrained environment, including canal preparation using available DHS instrumentation and the combination of a structural non-vascularized fibular strut with iliac crest structural and cancellous grafting as an alternative to techniques requiring microsurgical resources.

This study has several limitations, including the small sample size, heterogeneity in patient age, injury patterns, defect size, and injury severity, as well as the single-center and single-surgeon design and absence of a comparison group. These factors limit statistical inference, comparative assessment, and generalizability. The study was designed as a retrospective descriptive case series based on routine postoperative follow-up rather than a comparative study, and no control group was available to determine the relative effectiveness of this reconstruction compared with alternative techniques. Radiographic assessment was not blinded or independently adjudicated, and interobserver reliability was not assessed. Clinical assessment relied primarily on weight-bearing ability and range of motion, as validated functional outcome measures and formal donor-site outcome instruments were not feasible in the field-hospital setting. Formal nutritional assessment was also not consistently available, and serum nutritional markers, including vitamin D and calcium, were not systematically measured; although calcium and vitamin D supplementation was routinely provided, the influence of nutritional status on healing could therefore not be quantified. In addition, the follow-up period may be shorter than the potential refracture risk window. The lack of a clearly defined denominator for consecutive cases and the potential for survivorship bias should also be acknowledged, particularly in a conflict setting where patients unable to return for follow-up may differ systematically from those who remained available for assessment. The heterogeneity in age, ranging from 12 to 40 years, further limits direct comparison of outcomes across patients and should be considered when interpreting the findings. The unfavorable findings in this series also warrant consideration. Case 8 required staged reconstruction because of a pre-existing infected nonunion, highlighting that staged management may remain necessary when infection is present. Case 5 demonstrated a residual medial cortical gap despite substantial clinical and functional improvement, illustrating the limitations of defining healing solely on the basis of weight-bearing capacity. Limb-length discrepancy was observed in Cases 1 and 4, indicating that restoration of union does not necessarily ensure restoration of limb length. The mechanical failure in Case 8 occurred following premature progression to unrestricted weight-bearing and required revision fixation, emphasizing the importance of postoperative protection and adequate mechanical stability. Raised inflammatory markers in some patients further underscore the importance of infection assessment and control before definitive reconstruction. These findings indicate that the technique should not be interpreted as universally successful and that careful patient selection, infection control, mechanical planning, nutritional considerations, and longer follow-up remain important.

Overall, these findings highlight that successful limb salvage in complex long-bone defects may be facilitated by a balanced strategy integrating biological augmentation with adequate mechanical stability. The favorable experience with femoral defects in the present series, together with our previously reported experience with tibial nonunion and segmental bone loss in a war-related setting [19], suggests that combining NVFG with ICBG may represent a practical reconstructive option in selected patients, particularly in conflict-affected and resource-limited environments where advanced microsurgical techniques or prolonged staged reconstruction are not always feasible. The approach should, however, be individualized according to the presence of infection, soft-tissue status, defect characteristics, and mechanical requirements, with staged management undertaken when clinically indicated.

## Conclusions

Management of large post-traumatic femoral defects and established nonunion remains a complex reconstructive challenge, particularly in the presence of extensive bone loss, infection, and compromised biological conditions. In conflict-affected and resource-limited settings, access to advanced microsurgical and prolonged staged reconstructive procedures may be limited.

In this case series, reconstruction using NVFG combined with autologous ICBG provided a feasible limb-salvage strategy for large femoral segmental defects. Four patients experienced documented postoperative complications, and all eight patients ultimately achieved favorable union outcomes. These findings highlight both the potential of the technique to achieve union in challenging large femoral defects and the mechanical and functional challenges associated with such extensive reconstructions.

The approach may represent a practical reconstructive option in selected patients when microsurgical or prolonged staged reconstruction is not readily available. However, these preliminary findings from a small case series should be interpreted cautiously, and larger studies with longer follow-up are required to further evaluate union, mechanical complications, functional outcomes, and donor-site effects.
